# Stimulation of Sulfonamides Antibacterial Drugs Activity as a Result of Complexation with Ru(III): Physicochemical and Biological Study

**DOI:** 10.3390/ijms222413482

**Published:** 2021-12-15

**Authors:** Paulina Spisz, Agnieszka Chylewska, Aleksandra Królicka, Sandra Ramotowska, Aleksandra Dąbrowska, Mariusz Makowski

**Affiliations:** 1 Faculty of Chemistry, University of Gdańsk, ul. Wita Stwosza 63, 80-308 Gdańsk, Poland; agnieszka.chylewska@ug.edu.pl (A.C.); sandra.ramotowska@ug.edu.pl (S.R.); aleksandra.dabrowska@ug.edu.pl (A.D.); 2 Intercollegiate Faculty of Biotechnology of UG & MUG, University of Gdańsk, ul. Abrahama 58, 80-307 Gdańsk, Poland; aleksandra.krolicka@ug.edu.pl

**Keywords:** sulfonamides, sulfamerazine, sulfathiazole, metallodrugs, Ru(III) complexes, electrochemical profile, *CT*-DNA affinity, anticancer properties, antimicrobial properties

## Abstract

Antibiotic resistance is a global problem, and one promising solution to overcome this issue is using metallodrugs, which are drugs containing metal ions and ligands. These complexes are superior to free ligands in various characteristics including anticancer properties and mechanism of action. The pharmacological potential of metallodrugs can be modulated by the appropriate selection of ligands and metal ions. A good example of proper coordination is the combination of sulfonamides (sulfamerazine, sulfathiazole) with a ruthenium(III) ion. This work aimed to confirm that the activity of sulfonamides antibacterial drugs is initiated and/or stimulated by their coordination to an Ru(III) ion. The study determined the structure, electrochemical profile, *CT*-DNA affinity, and antimicrobial as well as anticancer properties of the synthesized complexes. The results proved that Ru(III) complexes exhibited better biological properties than the free ligands.

## 1. Introduction

**Figure 1:**
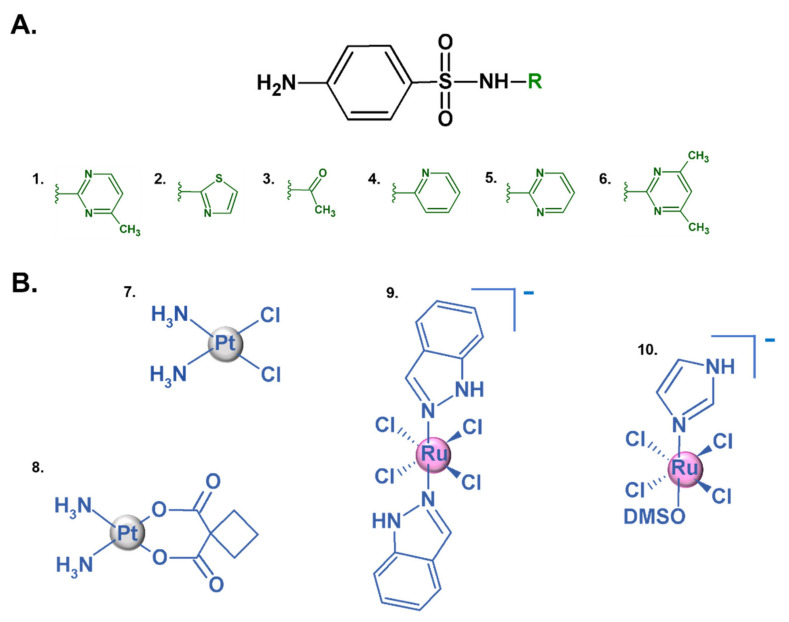
The general formula of sulfonamide-based antibacterial drugs and their examples: 1. sulfamerazine (SMZ), 2. sulfathiazole (STZ), 3. sulfacetamide, 4. sulfapyridine, 5. sulfadiazine, 6. sulfamethazine (**A**). Structures of the most well-known metal-containing drugs 7. cisplatin, 8. carboplatin, 9. NAMI-A, 10. KP1019 (**B**).

Modern antibiotic therapy has proven unsatisfactory in recent years. Antibiotics were first used as growth promoters in farm animals in the early 20th century, which led to their introduction into the daily human diet. As a result, a serious, previously unanticipated phenomenon emerged—the development of microbial resistance to antibiotics [1]. Antibiotic resistance accounts for 2.8 million infections in the USA every year. Unfortunately, more than 35,000 of these infections result in death [2]. Over the years, bacteria have developed resistance to a wide range of antibiotics, including sulfonamides (Figure 1A). These synthetic drugs are competitive inhibitors of dihydropteroate synthetase involved in the biosynthesis of tetrahydrofolic acid [3]. The widespread use of sulfa drugs in human and veterinary medicine, and their distribution in the environment have enabled bacteria to develop various strategies of resistance to these agents [4,5]. The main mechanism underlying sulfonamide resistance is target modification [3,4]. Moreover, sulfonamides can be degraded [6,7,8], modified [9], and even used as nutrients [10] by bacteria. In light of these facts, it seems extremely important to find a solution to antibiotic resistance.

Several combinations of metal ions and ligands—known as metallodrugs (Figure 1B)—have found different applications in pharmacology. The use of these compounds is considered to be a good alternative to traditional antibiotic therapy. Many studies have proven that metal complexes exhibit better pharmacological and toxicological properties, as well as a more effective mechanism of action, compared to free ligands [11,12]. Metallodrugs are characterized by excellent features, including unique geometry, changeable oxidation states and coordination numbers, and redox behaviors. In addition, metallodrugs can coordinate with various types of ligands, which makes them ideal for a wide range of applications in diagnostic and therapeutic medicine [13]. They have been successfully used as anticancer, antimicrobial, antiviral, and anti-inflammatory drugs. Moreover, metal-based agents can be used as adjuvants in immunotherapy to elicit an immune response [14,15,16]. Metallodrugs can interact with DNA, which is the main target in various therapies, including anticancer treatments. The interaction between metal-based drugs and DNA is either covalent or noncovalent. When the binding is covalent, the ligand can be displaced by the DNA base. In the case of noncovalent binding, there are three major possible modes of interactions: electrostatic, intercalative, and groove (surface) complex binding. All these interactions could cause chemical and conformational changes in the DNA structure [17,18,19].

The main examples of metal-based compounds are drugs based on platinum, lithium, silver, and bismuth [14]. However, ruthenium drugs also deserve attention. These drugs have unique electrochemical and spectroscopic properties [13,20,21]. Ruthenium is a good candidate for pharmacological applications because it exhibits various oxidation states (II, III, IV) under physiological conditions [13,22]. Ruthenium(III) complexes can be reduced to ruthenium(II) analogs in hypoxic conditions, and hence may act as prodrugs. Moreover, this ability makes them selective for cancer cells (hypoxia is characteristic for cancer cells in tumors) [23]. The therapeutic potential of metal ions is optimized by their connection to the core which is scaffolding. This not only tunes but also synchronizes the interaction of the ligand core with the target site [13]. Sulfonamides are an excellent choice of compounds for the core. These compounds are widely employed as antibacterial [24], antifungal [25], anticancer [26], and antioxidant [27] drugs. However, despite a large number of studies focusing on these metallodrugs and their well-known potential, the clinical application of these drugs is surprisingly limited [14].

Sulfamerazine (SMZ) (Figure 1A) is a sulfa drug characterized by antibacterial properties [28]. It is used in the treatment and prevention of many bacterial diseases such as infections of the eye, actinomics, urinary tract. Moreover, it has found applications in the treatment of prostatitis, bronchitis, meningitis [28,29]. Similar to other sulfonamides, SMZ may coordinate to a metal ion by nitrogen atom of the sulfonamide group and heterocyclic ring. The literature data show that in the case of sulfonamide derivatives also the oxygen of the sulfonamide group may be an active binding site [30]. Another main representative of sulfonamides, characterized by antibacterial properties is sulfathiazole (STZ) (Figure 1A), which is widely used in humans to treat topical skin infections and vaginal infections [31]. Other studies have shown that STZ can coordinate to metal ions by the nitrogen atom of the sulfonamide group and the heterocyclic ring [32].

The present work aimed to confirm that the activity of sulfonamides antibacterial drugs is initiated and/or stimulated by their binding to an Ru(III) ion. Two model systems, including four examined compounds, were selected for comparative analyses. The first system was sulfathiazole and [RuCl(OH_2_)(STZ)_2_]Cl_2_ · H_2_O complex **(1)**; and the second was—sulfamerazine and [RuCl_2_(SMZ)_2_]Cl complex **(2)**. Ru(III) coordination compounds were synthesized with STZ and SMZ and subjected to structural analyses. The electrochemical properties of the examined individuals were established. The interaction affinity of compounds to *Calf Thymus* *(CT)* DNA was determined, and the adequate values of binding constants were also established. The antimicrobial activity of the compounds was tested against both Gram-positive (G (+); *Staphylococcus aureus*, *Enterococcus faecalis*) and Gram-negative (G (–); *Pseudomonas aeruginosa*, *Escherichia coli*) bacteria, and fungi (*Candida albicans*). In addition, the cytotoxicity of the free ligands and Ru(III) complexes toward normal (HaCaT) and cancer cells (MCF-7, PC3) was investigated.

## 2. Results and Discussion

**Figure 2:**
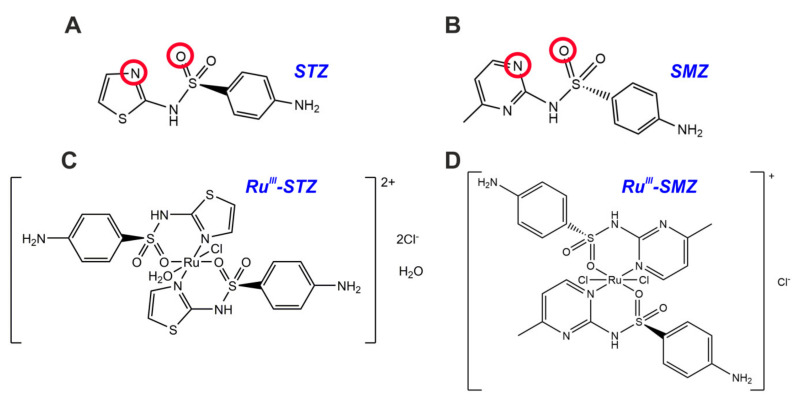
Structure of free ligands: (**A**) STZ, (**B**) SMZ (together with their donors’ atoms) and their Ru(III) coordination compounds: (**C**) [RuCl(OH_2_)(STZ)_2_]Cl_2_·H_2_O; (**D**) [RuCl_2_(SMZ)_2_]Cl emerged from the experimental analyses.

The selected STZ and SMZ sulfonamide derivatives were found to play two roles. The first one corresponds to their structures as representatives of the class of sulfonamides antibacterial drugs. This finding suggests that STZ and SMZ can be simple and useful models for improving the biological and chemical changes resulting from coordination with Ru(III) and the formation of adequate, stable complexes. The second role corresponds to their nature as sulfonamides ligands (Figure 2A,B) having an affinity for Ru(III) ions, which may allow creating a sulfamide-ruthenium drug (Figure 2C,D) with a significantly increased therapeutical index compared to the original sulfa-ligand.

Figure 2 shows the structure of ligands (SMZ, STZ) and their complexes with Ru(III). The data obtained as a result of the analyses described below prove that both sulfonamide ligands chelate the central ion through the nitrogen atom of the heterocycle ring and the oxygen atom of the –SO_2_NH– group.

Coordination of the metal ion by the N atom from the heterocycle and the O atom of the –SO_2_NH– group, occurring in the case of Ru(III)-STZ and Ru(III)-SMZ, is one of the known modes of complexations in such systems [33]. Sulfonamides however can coordinate with metal ions in different ways [30,33,34,35,36,37]. There could be found in the literature complexes with metal ions (e.g., Cu (II)) in which the coordination of the ion occurs only through the N atom of the thiadiazole ring [34], so the sulfonamide ligand, acts as a monodentate because neither the oxygen atom nor the nitrogen from the –SO_2_NH– group are involved in coordination. The complexation of the metal ion can also take place through *N*-heteroatoms and the N atom of the ligand –SO_2_NH– group [30]. Another mode of chelation can be binding of the central ion through the N atom of the aniline –NH_2_ group and the N atom of –SO_2_NH– [37]. There are also known complexes in which the coordination of the central ion [including Cu(II), Zn(II), and Co(II)] involves both the oxygen atom and the nitrogen atom from the –SO_2_NH– group [35], however, this is the case when the synthesis is carried out with the addition of NaOH to deprotonate/activate the nitrogen atom and the absence of heteroatoms in the ring associated with it.

### 2.1. Structural Analyses of New Ru^III^ Coordination Compounds

Generally, the presence of water molecules in compound **1** was confirmed by the results obtained from elemental, spectral, and thermal analyses. The coordination modes were established based on detailed spectral analyses (XRD, NMR). The presence of chloride counter ion(s) in Ru(III) complexes **(1)** and **(2)** was detected by adding a few drops of concentrated silver nitrate, which resulted in the appearance of a white precipitate, and by determining the conductivity values of the complexes **(1)** and **(2)**.

### 2.2. ATR Data to Structure Determination

The experimental (Attenuated Total Reflectance—ATR) and theoretical (IR) absorption bands obtained [RuCl(OH_2_)(STZ)_2_]Cl_2_ · H_2_O (**1**) and [RuCl_2_(SMZ)_2_]Cl (**2**) are depicted in Figure S1A,B, in the Supplementary Materials, respectively. The essential bands observed in the ATR spectra are presented in Table 1 and Figure S2. The theoretically calculated vibrational IR plots were reproduced in the experimental ATR spectra. This means that both methods are complementary and confirm the structural elements of the studied systems.

The IR spectra of the synthesized Ru(III) complexes **(1)** and **(2)** were similar to that of their sulfa drug precursors STZ and SMZ. However, some differences were observed in adequate IR spectra, which proves the successful complexation of Ru(III) ions. In the case of the free STZ sulfa ligand, a broad absorption band observed at 3271 cm^−1^ indicating the presence of -NH moiety of sulfonamide group was moved by 171 cm^−1^ to lower wavenumbers for **(1)**. This means that the functional group was involved in the binding of the Ru(III) ionic center. The stretching vibrations of NH (in aromatic amine moiety) in STZ for STZ at 3351 and 3319 cm^−1^ were noted at slightly higher wavenumbers (3470 and 3368 cm^−1^, respectively) on the spectrum of compound **(1)**. These changes prove that the amine group (in aniline residue) was not involved in the coordination of Ru(III), and also confirm the presence of water and that it was coordinated to structure **(1)**. Interestingly, the same coordination mode was suggested by the IR (by ATR method) data analysis for compound **(2)**, excluding the presence of aqua ligand in the coordination sphere. The lower shifts of symmetric and asymmetric vibration of amino (in aromatic moiety) group showed that NH_2_ residue did not participate in the chelation process of metal ions. Additionally, the –SO_2_ band of the sulfonamide group –SO_2_NH– was observed at 1324 cm^−1^ (asymmetrical) for the free SMZ sulfa drug, but it shifted to a lower wavenumber by 22 cm^−1^ upon complexation [38]. All these changes prove that the –SO_2_ sulfonamide group (O donor atoms) was involved in the coordination of Ru(III) ion in both complexes **(1)** and **(2)**. Finally, the new medium-to-weak bands observed in the IR spectra of both Ru(III) complexes in the range of 458–448 cm^−1^ can be assigned to ν(Ru-N) bands [39,40]. This confirms that the *N*-heteroatoms of sulfa ligands were involved in the chelation of Ru ions. The theoretical values were found to be slightly shifted in comparison to the experimental ones. However, in general, the theoretical and experimental values were in good agreement.

### 2.3. NMR Investigation

^1^H NMR provided important structural information for Ru(III) and sulfonamide coordination compounds [41]. The ^1^H and ^13^C NMR spectral data of Ru(III) complexes together with selected sulfa derivatives were recorded in DMSO-d_6_. The intensities of all resonance lines are presented in Figures S3–S6. The characteristic signals were assigned by comparing them with the spectra of similar Ru(III) complexes [42]. The ^1^H NMR spectra of **(1)** and **(2)** had several signals that corresponded to Ru(III) metal ions exhibiting paramagnetic properties. Moreover, multiplicity loss observed on the spectra of complexes can also be attributed to the proximity of the paramagnetic center. The integration values were in agreement with the proposed structure (assignment data are included in the “Materials and Methods” section). The positions of protons corresponded well to the proposed structure of Ru(III) complexes and were assigned based on the earlier reports [43,44]. Moreover, both types of NMR spectra indicated the high purity of the studied samples.

### 2.4. X-ray Diffraction Studies

The X-ray diffraction patterns of the obtained Ru(III) complexes are shown in Figure S7. Due to the amorphous phase of [RuCl(OH_2_)(STZ)_2_]Cl_2_·H_2_O **(1)**, the pattern of this complex was of low intensity with no well-defined peaks. However, two peaks were taken into consideration for determining the probable size of its crystallites (Table S1). On the other hand, the diffraction pattern of the [RuCl_2_(SMZ)_2_]Cl **(2)** complex was well-defined, sharp, and of high intensity, indicating that it is a crystalline phase. Based on the peak width at half of the height of the most intense peak at 2θ = 26.0, the total average crystallite size of complex **(2)** was estimated to be 29 nm, by applying the Debye–Scherrer equation [45,46]. In the case of compound **(1)**, the crystallite size determined was 1.30 nm, which implies that the nanoform was its powder.

### 2.5. UV-Vis Analysis and Electrical Conductance

**Figure 3:**
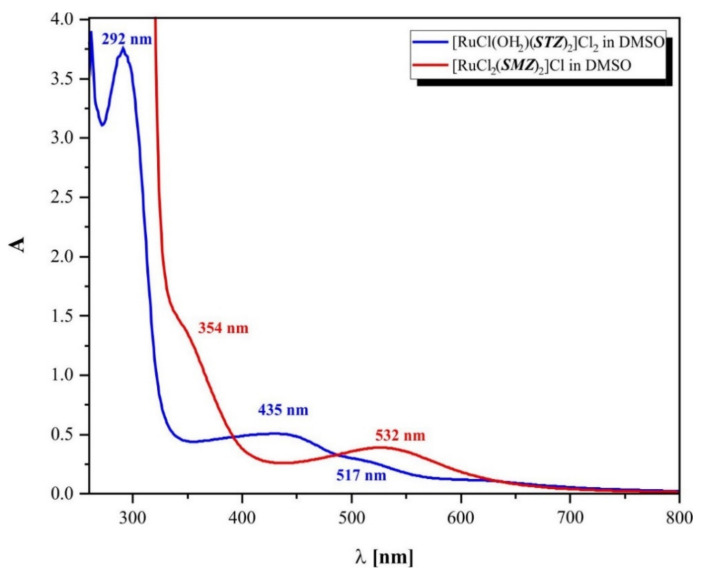
UV-Vis spectra of: (blue line) [RuCl(OH_2_)(STZ)_2_]Cl_2_·H_2_O (1); (red line) [RuCl_2_(SMZ)_2_]Cl (2) complexes’ solutions (DMSO).

The electronic spectral peaks of the STZ and SMZ free ligands were observed in a range of 200–400 nm (described in the “Interaction affinity to *CT*-DNA biomolecule” subsection). The data provided refer to the two absorption bands at 204 and 261 nm obtained with highly diluted solutions of **(1)** and **(2).** These bands corresponded to π–π* transitions attributed to the aromatic rings [47] of the sulfonamide ligands. The spectra registered for both complexes in the visible region were dominated by the expected d–π* MLCT transitions (435 and 517 nm for compound **(1)**; 532 nm for compound **(2)**). The electronic spectral peaks of both complexes **(1)** and **(2)** (Figure 3) suggested an octahedral geometry with slight distortion. The ground states of Ru(III) complexes had ^2^T_2g_ arising from the (t_2g_)^5^ configuration in an octahedral **(2)** geometry (Figure 1). Moreover, the DMSO solutions of both **(1)** and **(2)** were colored, which is typical for this type of environment (Figure S8).

As the synthesized complexes were poorly soluble in water at 25 °C, the conductivity values of their 1 mM solutions were measured in a water and methanol mixture (*v*/*v* = 1:1; 2.42 µS∙cm^−1^). The conductivity of STZ and SMZ free ligands was measured to be 3.27 and 2.57 µS·cm^−1^, respectively. Based on these values, the sulfonamide ligands were classified as nonelectrolytes. In turn, the conductivity values determined for Ru(III) complexes **(1)** and **(2)** (Table 2) indicated that they were electrolytes. The conductivities of 1 mM solutions of NiCl_2_ and NH_4_Cl were also measured in the water and methanol, and the values were 123.15 and 12.91 µS∙cm^−1^, respectively, which were approximate to the values of the synthesized complexes. This means that the examined coordination compounds are of 1:2 and 1:1 electrolyte types similar to NiCl_2_ and NH_4_Cl solutions, respectively. These results confirmed that one or two chloride anions were counter ions in the coordination spheres of the studied ion complexes [48].

### 2.6. TG-DTG Analysis

The results of the thermal assay analysis revealed the different courses of the decomposition process when the free ligands (STZ or SMZ) and adequate ruthenium coordination compounds **(1)** or **(2)** were compared in pairs. The data obtained from TG-DTG analysis were helpful in structure determination. The hydration water, compared to the water inside the sphere, can be easily recognized by the lower temperature of its loss by the studied complexes. Although the present work aimed to analyze the decomposition of sulfa drugs STZ and SMZ, they were treated as a standard to compare their thermal behavior with that of the newly synthesized ruthenium complexes **(1)** and **(2).** The thermal decomposition of the [RuCl(OH_2_)(STZ)_2_]Cl_2_·H_2_O **(1)** complex involved three main steps. However, the second and third steps of decomposition can be further divided into substeps. These were directly related to the loss of a water molecule from the inside sphere as well as the release of chlorine. The first stage of decomposition of **(1)** occurred at 98 °C and was accompanied by the formation of an anhydrous complex [RuCl(OH_2_)(STZ)_2_]Cl_2_. This intermediate product further decomposed to yield the final product, RuS_2_, and residual carbons. The total weight loss was estimated at 69.90% on TG for complex **(1)**, and the amount of dry residue was established to be 30.01%. The detailed TG-DTG data and percentage mass losses are presented in Table 3.

In the case of [RuCl_2_(SMZ)_2_]Cl **(2)**, the TG-DTG analysis proved that the chlorides were coordinated to the Ru(III) center. The compound showed good thermal stability, with no weight loss up to 190 °C. The total weight loss was estimated at >74.99% on TG, and the amount of dry residue was equal to 25.01%.

It should be mentioned that the theoretically calculated loss of each fragment or specific molecule corresponded very well to the values determined in the experiments, where the objects were studied as a whole. The complete thermograms are shown in Figures S9–S12.

### 2.7. Electrospray Ionization Mass Spectrometry Analysis (ESI-MS)

Mass spectrometry is a powerful tool that has been increasingly applied in coordination chemistry for structural characterization [49,50,51]. The ESI-MS spectra of Ru(III) complexes dissolved in methanol are presented in Figure S13. The mass spectra of the studied compounds were recorded in the negative mode in the m/z range of 50–1000. The main molecular peaks were observed at m/z of 753.6, 253.8, and 197.6 for Ru(III)-STZ, and at 735.7, 697.8, and 262.9 for Ru(III)-SMZ, respectively, with the first values corresponding to the actual molecular weights of these complexes. It is worth noting that the MS peaks observed on the spectra of complexes **(1)** and **(2)** exhibited the correct distribution of isotopomers mainly derived from the Ru ion. The results of ESI-MS analysis of each of the Ru(III) complexes containing sulfonamide derivatives supported the proposed structure of the coordination compounds. Moreover, the experimental data obtained for all the studied Ru(III) compounds are in good agreement with and correspond well to a previous report on a similar type of complexes [47].

### 2.8. Interaction Affinity to CT-DNA Biomolecule

**Figure 4:**
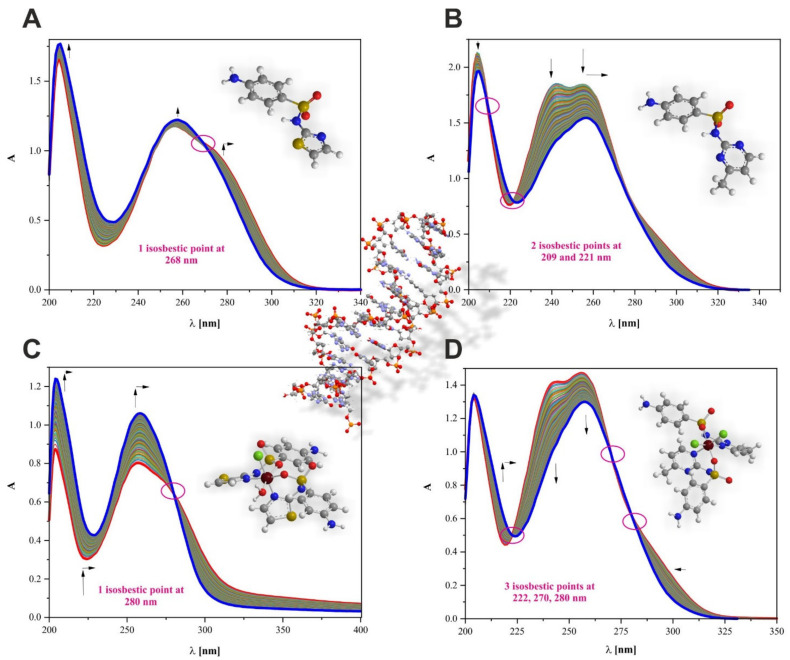
Changes observed in absorption spectra of (**A**) STZ (14 μM), (**B**) SMZ (66 μM), (**C**) [RuCl(OH_2_)(STZ)_2_]Cl_2_·H_2_O (28 μM), (**D**) [RuCl_2_(SMZ)_2_]Cl (80 μM) solutions. These spectra were registered in 5 mM Tris-HCl (50 mM NaCl) buffer pH 7.39 in the presence of different concentrations of DNA from 0–114 μMbp. The absorbance changes at selected wavelengths were included in Supplementary Materials Figure S15.

The interaction of Ru(III) and sulfonamide complexes **(1)** and **(2)**, as well as STZ/SMZ, unbound ligands with DNA caused absorbance changes and wavelength shifts. The spectra of the compounds titrated with *CT*-DNA are shown in Figure 4. The experiment clearly showed an interaction through binding with the major groove mode because it caused hypochromic or hyperchromic and red shifts due to the stacking effect of DNA base pairs and an aromatic chromophore of the compounds STZ, SMZ, **(1),** and **(2)**. The presence of isosbestic points in the UV spectra (for all samples) indicated simple equilibria between unbound and bound molecules (Figure S14). The most significant changes in the maxima positions and absorbance values upon the addition of DNA were observed for complexes **(1)** and **(2)**.

The results proved the higher affinity of Ru(III) sulfonamide complexes for the studied biomolecule than for free STZ and SMZ. The interaction with DNA was directly related to the ligands inside the coordination sphere **(1)** and **(2)**, and therefore, measurements in the UV region were performed with diluted solutions of Ru(III) complexes. The preliminary investigations verified the visible range of registration (excluded) for this type of interaction (Figures S15 and S16).

This study showed that the [RuCl(OH_2_)(STZ)_2_]^2+^ complex was characterized by a higher binding constant compared to the STZ ligand, which was confirmed by the values of intrinsic binding constants K_b_ = 2.01·10^4^ and K_b_ = 3.10·10^5^ for ligand STZ and complex [RuCl(OH_2_)(STZ)_2_]^2+^, respectively (Figure S16A,C). The K_b_ values were calculated using the Wolfe–Shimer equation (Equation (1)) [52]:

(1) $$\frac{\left[\mathrm{DNA}\right]}{\varepsilon_{a}{-\varepsilon }_{f}}=\frac{\left[\mathrm{DNA}\right]}{\varepsilon_{b}{-\varepsilon }_{f}}+\frac{1}{K_{b}\left(\varepsilon_{b}{-\varepsilon }_{f}\right)}$$

where [DNA] is the concentration of *CT*-DNA and ε is the appropriate extinction coefficient. The STZ molecule, and especially the free anilino moieties, played a key role in the process of binding [53], which was also proven by their similar binding constants. A detailed comparison of interactions showed that the main band at 256 nm showed changes, with a 2 nm bathochromic shift with 25% hyperchromicity observed for compound **(1)** as a result of DNA addition. On the contrary, the position of the band at 256 nm corresponding to the free form of STZ changed by a 2 nm red shift upon the formation of the DNA-STZ adduct. However, this effect was accompanied by hypochromicity of almost 4% (Figure 4A,C). Interestingly, the single isosbestic point was observed at 268 nm for the DNA-STZ system and 280 nm for the DNA-[RuCl(OH_2_)(STZ)_2_]Cl_2_ system. The difference between their positions was directly related to the presence of a trivalent Ru ion stabilizing the skeleton of the STZ ligands after binding.

Similarly, the [RuCl_2_(SMZ)_2_]^+^ complex was characterized by a slightly higher binding constant compared to the SMZ ligand, as confirmed by the values of intrinsic binding constants K_b_ = 1.51·10^4^ and K_b_ = 7.03·10^4^ for ligand SMZ and complex [RuCl_2_(SMZ)_2_]^+^, respectively (Figure S16B,D). A detailed comparison of interactions showed that the main band at 256 nm showed changes, with a 2 nm bathochromic shift with 12% hypochromicity observed for compound **(2)** as a result of DNA addition. On the contrary, the position of the band at 254 nm corresponding to the free form of SMZ changed by a 2 nm red shift upon the formation of the DNA-*SMZ* adduct. However, this effect was accompanied by 17% hypochromicity (Figure 4B,D). Interestingly, two isosbestic points were observed for DNA-SMZ, at 209 and 221 nm, and three for the DNA-[RuCl_2_(SMZ)_2_]Cl system, at 222, 270, and 280 nm. The differences were related to the trivalent Ru ion stabilization of the SMZ ligand skeletons after binding.

### 2.9. Fluorescence Quenching Study

**Figure 5:**
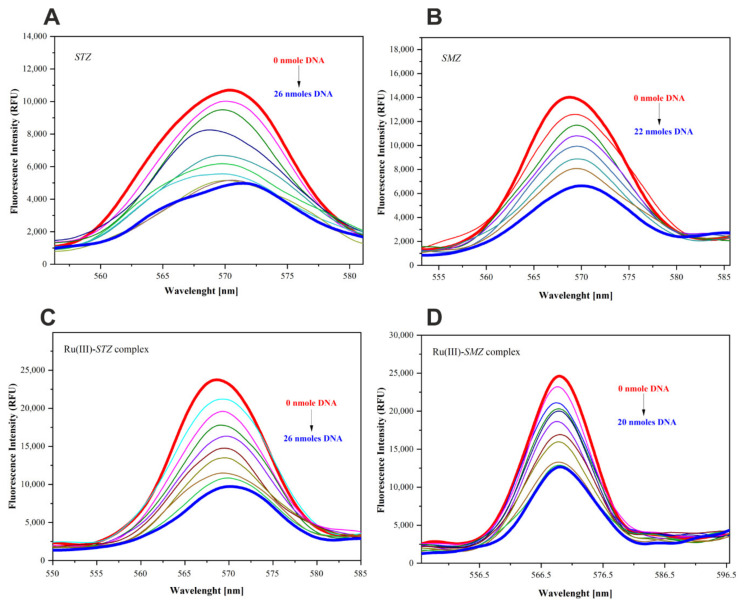
Changes observed in fluorescence emission spectra of (**A**) SMZ (18 μM), (**B**) STZ (24 μM), (**C**) Ru(III)-SMZ complex (69 μM), and (**D**) Ru(III)-STZ complex (146 μM), with increasing of *CT*-DNA given in nmoles, (λ_exc_ = 260 nm, λ_em_ = 570 nm).

Fluorescence spectroscopy is an ideal method for studying the interactions occurring between small-molecule ligands and a biomacromolecule. A vast amount of information about the interaction can be collected by measuring parameters such as emission peaks, energy transfer efficiency, and lifetime. The effects of *CT*-DNA on the fluorescence intensity of SMZ, STZ, Ru(III)-SMZ, and Ru(III)-STZ are illustrated in Figure 5. It was noted that the fluorescence intensity of the studied compounds decreased regularly as the concentration of *CT*-DNA increased. This effect is referred to as fluorescence quenching, and it may result from various processes such as excited-state reactions, ground-state complex formation, and collisions. Static quenching occurs due to the formation of a ground-state complex between the fluorophores and the quencher. On the other hand, collisional quenching or dynamic quenching results from collisions between the fluorophores and the quencher, and can be mathematically expressed by the Stern–Volmer equation (Equation (2)):

(2) $$\frac{F_{0}}{F}=1+K_{\mathrm{SV}}\left[Q\right]=1+k_{q}\tau_{0}\left[Q\right]$$

where F_0_ and F are the fluorescence intensities of sulfonamides in the absence and presence of the quencher, respectively. K_q_ is the quenching rate constant of sulfonamides, K_sv_ is the dynamic quenching constant, τ_0_ is the average lifetime of the molecule without the quencher, and [Q] is the concentration of the quencher.

The graphs plotted according to the above equation (Equation (2)) are shown in Figure S17. The static quenching constants, K_SV_, were calculated based on the slope of regression curves in the linear range.

The K_SV_ values determined from the plot F_0_/F vs. [Q] (Figure S17) are listed in Table 4. Those values with a magnitude order of 10^3^ M^−1^ were considered to be indicative of a strong interaction between DNA and metal complexes [54,55,56].

### 2.10. Association Constants and the Number of Binding Sites

Based on the results, it can be postulated that the fluorescence quenching of *CT*-DNA is a static quenching process, which can be mathematically expressed by the Lineweaver–Burk formula (Equation (3)) [57]:

(3) $$\frac{1}{F_{0}-F}=\frac{1}{F_{0}}+\frac{K_{D}}{F_{0}\left[Q\right]}$$

where K_D_ is the dissociation constant, [Q] is the concentration of the quencher, and F_0_ and F are the fluorescence intensities of sulfonamides in the absence and presence of the quencher.

The Lineweaver–Burk double-reciprocal plots were constructed based on the relationship of (F_0_/F) vs. various concentrations of *CT*-DNA (Figure S18). From the regression equation of curves, the values of association constants (K_A_ = 1/K_D_) for interaction between the studied compounds and *CT*-DNA were determined (Table 4).

It was noted that the association constant values were high, which indicates that *CT*-DNA had a high affinity to the studied compounds. The order of affinity of the studied compounds was as follows: STZ < Ru(III)-SMZ < SMZ < Ru(III)-STZ. The Scatchard equation (Equation (4)) provided below can be used to estimate the number of binding sites between an organic micromolecule and a biological macromolecule based on the above-stated conclusion that fluorescence quenching is caused by static quenching resulting from the compound formation:

(4) $$\log\left[\frac{F_{0}-F}{F}\right]={\mathrm{logK}}_{A}+\mathrm{nlog}\left[Q\right]$$

where $K_{A}$ represents the static association constant, n is the number of binding sites, $\left[Q\right]$ is the concentration of the quencher, and F_0_ and F are the fluorescence intensities of sulfonamides in the absence and presence of the quencher. The plots were constructed based on the relationship of log[(F_0_ − F)/F] and log[Q] (Figure S19). From the regression equation of curves, the association constants (K_A_) and number (n) of binding sites were calculated. The results showed that the obtained values of association constants were following those calculated using the above-described Lineweaver–Burk equation. It was noted that in all the studied compounds the number of binding sites involved in interaction with *CT*-DNA was 1 (Table 4). This indicates that the binding stoichiometry of sulfonamides and their Ru(III) complexes with *CT*-DNA molecule was 1:1.

### 2.11. Electrochemical Profile

The analysis of the electrochemical properties of compounds can provide information on their redox forms in a solution [58]. Establishing the physicochemical profile of compounds with potential biological uses is extremely important for both understanding their mode of action and determining the most favorable conditions to achieve maximum pharmaceutical efficacy [59]. Knowledge of the redox properties of pharmaceuticals is also of use in studies focusing on developing new methods that allow electrochemically controlled drug release [60]. Moreover, it has been shown that the mechanism of the biological activity of some compounds is influenced by their redox activity [61,62]. Reliable and accurate correlations between the electrochemical properties and the biological activity of pharmaceuticals are still being sought. Thus, the determination of the redox profile is important in the study of new substances.

**Figure 6:**
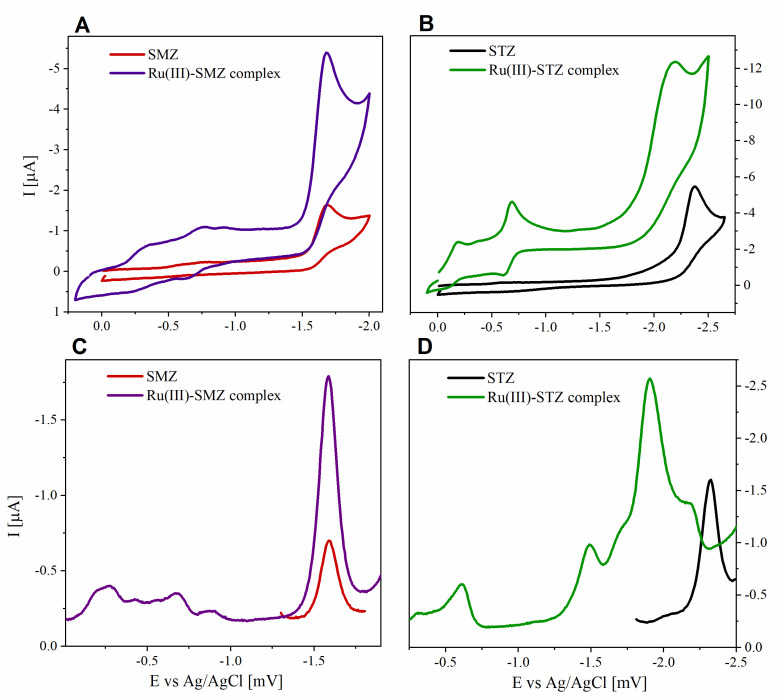
Voltammograms registered for studied ligands and complexes (concentration of 2·10^−3^ M) in DMSO (**A**) CV of SMZ and Ru(III)-SMZ complex (**B**) CV of STZ and Ru(III)-STZ complex (**C**) DPV of SMZ and Ru(III)-SMZ complex (**D**) DPV of STZ and Ru(III)-STZ complex.

The studied ligands placed in an aprotic solvent (DMSO) underwent electroreduction (red and black lines in Figure 6A,B). The measurements were performed at a scan rate of 100 mV·s^−1^. The SMZ cathodic peak was located at a more positive potential (E_c_ = −1.689 V) than STZ (E_c_ = −2.377 V), which proves that the reduction of the SMZ molecule occurred more easily. The voltammograms registered with CV showed no anodic response, indicating the irreversibility of the cathode process. Differential pulse voltammograms were also recorded for sulfonamide ligands (red and black lines in Figure 6C,D). The reduction signals measured with the DPV technique were consistent with the values of cathodic peak potential determined from CV (Table 5).

Regarding the electrochemical characteristics of ligands in an aqueous medium, *SMZ* underwent electro-oxidation at a potential of approximately 0.9 V and the signal was dependent on the pH of the solution [63]. By contrast, STZ showed very low electrochemical activity, which was also registered in water and Tris buffer (pH 7.4) with the addition of 5% DMSO (Figure S20).

The comparative analysis of ruthenium complexes about sulfonamide ligands aimed to confirm the formation of coordination compounds, determine their electrochemical properties, and demonstrate the differences between the electrochemical profile of ligands and the newly synthesized complexes. Generally, Ru(III) complexes are relatively inert toward living cells and can be reduced to active forms of Ru(II) in an environment with a lower pH. This property is valuable for the application of ruthenium complexes as anticancer agents [20]. Due to increased metabolism and thus reduced oxygen concentration, cancer cells have a higher level of glutathione (GSH) and a lower pH than normal cells. As a result, a strong reducing environment is created within the tumor cell, which can lead to the activation of Ru(III) ions to Ru(II) form [64]. Thus, modification of the redox potential may allow increasing the selectivity of action and reduce the toxicity of a drug. A comparison of cyclic voltammograms recorded for sulfonamides and their complexes showed an increase in the intensity of the signal from the ligand moiety, which was consistent with the stoichiometry of the coordination compound (Figure S20A,B). For the Ru(III)-SMZ complex, the reduction signal from the ligand remained at a practically unchanged potential value (E_c_ = −1.689 and −1.677 V for SMZ and the [RuCl_2_(SMZ)_2_]^+^ complex, respectively; ΔE = 12 mV). However, in the case of the Ru(III)-STZ complex, a shift of the reduction peak toward more positive potentials was observed (E_c_ = −2.377 and −2.202 V for SMZ and [RuCl_2_(SMZ)_2_]Cl **(2)**, respectively; ΔE = 175 mV). This indicates an easier reduction of the sulfonamide unit in the coordination compound as compared to the free ligand. Moreover, for complexes, new signals were observed in the area of less negative potentials, unlike the voltammograms recorded for ligands. The new peaks were related to the reduction of Ru(III) ions in the coordination center to the Ru(II) form. These peaks were more distinct for the STZ complex **(1)**. In this case, two peaks were observed—a small one at E_c_ = −0.188 V and a more intense one at E_c_ = −0.688 V. The signal at the value of E_c_ = −0.688 V was accompanied by a developed anode response, and the oxidation peak was observed at E_a_ = −0.600 V. For compound **(2)**, the new signal was of very low intensity and observed at E_c_ = −0.751 V.

**Figure 7:**
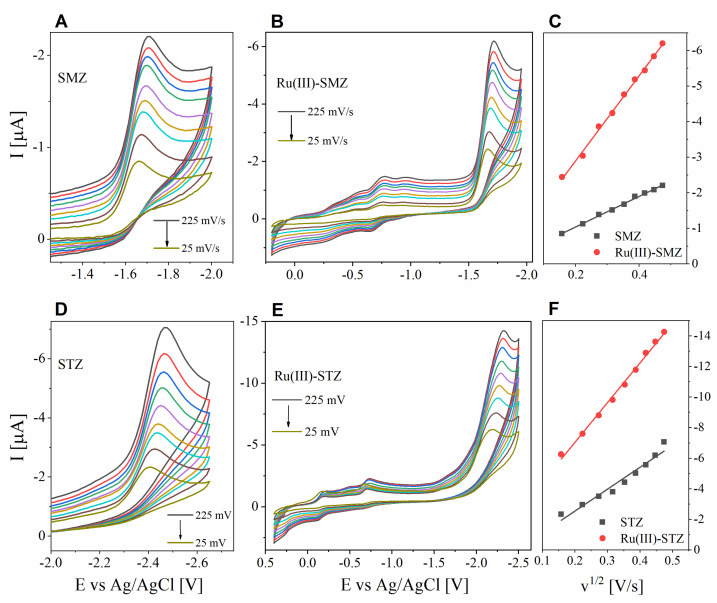
Voltammograms in DMSO registered for (**A**) SMZ, (**B**) Ru(III)-SMZ, (**D**) STZ, (**E**) Ru(III)-STZ, registered at different values of scan rate (from 25 mV·s^−1^ to 225 mV·s^−1^). Plots of reduction current I vs. v^1/2^ for (**C**) (■) SMZ (r^2^ = 0.998) and (●) Ru(III)-SMZ (r^2^ = 0.999) and (**F**) (■) STZ (r^2^ = 0.950) and (●) Ru(III)-STZ (r^2^ = 0.994).

In the voltammetric methods, the influence of the scanning speed (ν) on the course (i.e., position and intensity of redox signal) is an important diagnostic criterion for explaining the type of mechanism. To elucidate the type of process, the values of the current response (I) were registered for both ligands (Figure 7A,D) and ruthenium complexes (Figure 7B,E) at different speeds (ν) of the potential sweep (from 25 to 225 mV·s^−1^, an interval of 25 mV). For all the studied compounds, with an increase in scan rate, a proportional increase in signal intensity was observed, with a simultaneous shift toward more negative potentials. The change in scanning speed did not have any impact on the reversibility of the process. The current response dependence on the square root of ν was determined (Figure 7C,F). The peak currents signals from ligand unit redox processes were found to vary linearly with the square root of the scan rate (ν^1/2^). This indicates that the registered electrochemical processes were, as expected, diffusion-limited.

Based on the recorded cyclic voltammograms, the values of diffusion coefficient (D) were determined (Table 5) for the tested compounds using the Randles–Ševcik equation (Equation (5)) for irreversible electrochemical processes [65,66]:

(5) $$I_{p}=0.496\;nFAC\sqrt{\frac{\alpha nFvD}{RT}}$$

where $I_{p}$ is the voltammetric current, n is the number of electrons in the electrochemical reaction, F is the Faraday constant [C·mol^−1^], A is the electroactive area of the electrode [cm^2^], C is the concentration [mol·cm^−3^], α is the transfer coefficient, v is the applied voltammetric scan rate [V·s^−1^], D is the diffusion coefficient [cm^2^·s^−1^], R is the gas constant [J·K^−1^·mol^−1^], and T is the temperature [K]. For ligands, a one-electron reduction reaction was adopted, while for coordination compounds (due to the complex cation structure with two ligand molecules), a two-electron reduction process was adopted. The diffusion coefficient of the electroactive substance, which refers to the speed at which the depolarizer molecules move to the electrode surface, is a characteristic criterion of a given substance under specific measurement conditions, as well as an important parameter for assessing the interaction of the tested compound, for example, with biomolecules. In the case of both the pairs of systems compared in the study, the D values of the coordination compounds were found to be lower than that of the corresponding ligands, which is consistent with the size of the depolarizer molecules due to the slower movement of larger particles in the solution [67].

### 2.12. Antimicrobial Activity—Minimal Inhibitory Concentration

The synthesized [RuCl(OH_2_)(STZ)_2_]Cl_2_ · H_2_O **(1)** and [RuCl_2_(SMZ)_2_]Cl **(2)** complexes were tested for their antimicrobial activity. It was observed that Ru(III)-STZ showed higher antimicrobial activity against two representatives of G (+) bacteria and two representatives of G (–) bacteria (Table 6). Moreover, with Ru(III) complex **(1)**, it was possible to reduce the antibiotic dose by 33% for all the studied organisms. This is a significant result because Munteanu and Uivarosi [68] recently showed that ruthenium complexes, in general, exert excellent activity against Gram-positive bacteria (e.g., *S. aureus*) and, display lower activity towards Gram-negative bacteria (e.g., *E. coli* and *P. aeruginosa*). In the case of the application of the complex (1), the antimicrobial activity against Gram-negative bacteria concerning the pure sulfonamides antibacterial drugs (STZ) increased by 1/3. When a different Ru(III)-SMZ complex was used, a similar result was observed only for *E. coli* and *E. faecalis* (Table 6), and the increase in antibacterial activity was 28%. The dangerous ESCAPE pathogens, *S. aureus* and *P. aeruginosa* [69], remained insensitive to the applied doses of the pure sulfonamides antibacterial drugs (SMZ) and complex **(2)** with Ru(III). Sulfonamides interrupt the synthesis of folic acid in bacterial cells, by competitively inhibiting the reaction between p-aminobenzoic acid and dihydropteridine. This leads to the inhibition of the synthesis of thymidine (essential for DNA synthesis), purines, and some amino acids [70]. The results indicated that the obtained complex showed increased antimicrobial activity due to the combination of Ru with STZ. This may be related to the fact that the penetration of the antibiotic through the lipid cell membrane was higher, and that the metal-binding sites in the enzymes of the microorganisms were blocked [37]. Another possible explanation may be that Ru(III) complexes can interact with DNA via both covalent and noncovalent interactions and with RNA and protein [71].

### 2.13. Cytotoxicity Assay

The main purpose of designing new metallodrugs is to create compounds with new or better properties, compared to the free ligands. To verify the hypothesis that complexation of compounds with metal ions will lead to new complex ([RuCl(OH_2_)(STZ)_2_]Cl_2_·H_2_O **(1)** and [RuCl_2_(SMZ)_2_]Cl **(2)** with increased anticancer activity of toward cancer cells (MCF-7 and PC3) the WST-1 assay was performed. Additionally, to compare the anticancer activity of the new complexes with that of the free ligands, the same test was carried out for STZ and SMZ. Moreover, these results were compared with those obtained for normal cells (HaCaT).

**Figure 8:**
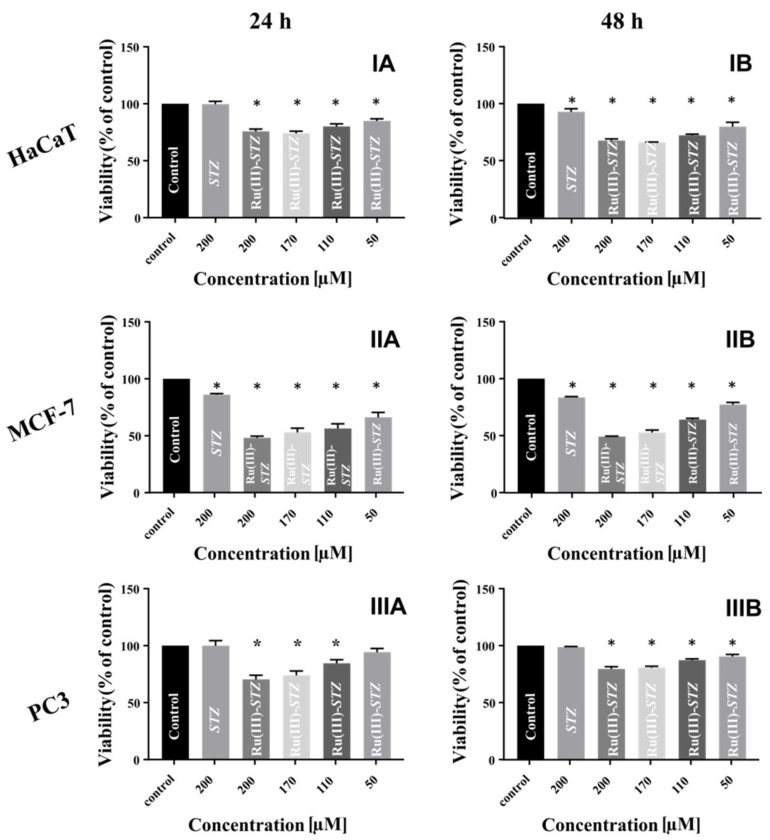
The viability of I. HaCaT, II. MCF-7 and III. PC3 cells after: (**A**) a 24 and (**B**) a 48 h treatment with STZ and Ru(III)-STZ complex in the range of concentration from 0 (control) to 200 µM. Results are shown as mean ± SD of three independent experiments performed in triplicate. * Statistically significant difference is present between treated cells compared with untreated cells (control).

STZ and SMZ were tested at a concentration of 200 µM, whereas complexes **(1)** and **(2)** were tested at four different concentrations (50, 110, 170, and 200 µM) and with two incubation times (24 and 48 h). Untreated cells were used as a control. The results obtained for all three cell lines after 24 and 48 h of incubation with STZ and [RuCl(OH_2_)(STZ)_2_]Cl_2_ · H_2_O **(1)** complex are shown in Figure 8. Compared to the free ligand STZ, treatment with Ru(III)-STZ complex significantly reduced viability in all three cell lines. The highest reduction in viability was observed for the MCF-7 cell line. In this case, the statistically significant reduction in viability after 24 h was equal to 48.14 ± 1.24 for a concentration of 200 µM, while incubation with STZ at the same concentration reduced viability to 86.11 ± 0.79. Most importantly, these results showed an increase in the anticancer activity of 45% for complex **(1)**. After 48 h, a similar effect was observed. The significant reduction of viability allowed determining the IC_50_ values of the studied compounds. For the Ru(III)-STZ complex, the IC_50_ values were equal to 183 and 187 µM, respectively, for 24 and 48 h incubation, this means that viability is independent of the incubation time with a compound.

SMZ and its [RuCl_2_(SMZ)_2_]Cl **(2)** complex also caused a reduction in viability (Figure S21) in HaCaT and the MCF-7 cells lines with both time variants, but in this case, the reduction was moderate. The difference in action between the free ligand and the complex was also not as marked as in the case of STZ. It is worth emphasizing that both the STZ and the SMZ ligand were tested at almost threefold higher concentrations (590 µM for STZ and 570 µM for SMZ), but even in these concentration variants, the viability reduction was not found to be lower than that in the case of complexes tested at 200 µM concentration. These data confirm that complexation, especially of STZ, with Ru(III) ions, improves anticancer properties. This finding encourages further research on the mechanism of action of ruthenium drugs.

## 3. Materials and Methods

### 3.1. Materials

The standard precursors of sulfonamides antibacterial drugs, sulfathiazole (STZ; white powder; ACS reagent; 98%) as well as sulfamerazine (SMZ; white powder; ACS reagent; 98%) were purchased in pure form from Sigma-Aldrich company (Saint Louis, MO, USA). Ruthenium(III) chloride hydrate of analytical grade (40–43% Ru) (99.9% -Ru) was obtained from Strem Chemicals Inc (Newburyport, MA, USA). and used without further purification. The following chemicals were also of analytical grade and purchased from Sigma-Aldrich: Tris(hydroxymethyl)aminomethane (Tris; solid; ACS reagent, ≥99.8%,), HCl (36.5–38%; liquid; bioreagent for molecular biology), tetrabutylammonium perchlorate (TBAP; solid; ACS reagent, ≥99%), perchlorate sodium monohydrate (NaClO_4_; solid; ACS reagent, 98%). The *CT*-DNA was purchased from the Sigma-Aldrich company. Cell proliferation reagent (WST-1) and antibiotics (solution stabilized, with 10,000 units penicillin and 10 mg streptomycin∙mL^−1^) were purchased from Sigma-Aldrich. HDFa, F-12K, DMEM, and fetal bovine serum (FBS) were obtained from Gibco (Billings, MT, USA). The bacteria strains G (+): *S. aureus* ATCC 25923, *E. faecalis* ATCC 19433 as well as G (−): *P. aeruginosa* ATCC 27853, *E. coli* ATCC 25922, and fungi *C. albicans* ATCC 90028 were obtained from the American Type Culture Collection (ATCC) (Manassas, VA, USA). A commercial antibiotic: ciprofloxacin—fluoroquinolone (KRKA, d.d., Novo Mesto, Slovenia) was used for comparative purposes. Twice distilled water (Hydrolab-Reference purified) with a conductivity below 0.08 µS∙cm^−1^ was used for preparing all the solutions.

### 3.2. General Methods

The percentage elemental composition (CHNS) of the synthesized compounds was determined using a Carlo Erba EA 1108 CHNS element analyzer. Infrared spectra of Ru(III) complexes, as well as substrates, free sulfa ligands, and Ru(III) chloride, were recorded using the ATR technique with a Spectrum Two spectrometer (PerkinElmer, Waltham, MA, USA) in the range of 4000–400 cm^−1^. Theoretical IR signals for the systems studied in this work were obtained using the procedure proposed by Minić et al. [72]. The XRD powder diffractograms of two Ru(III) sulfa complexes were recorded using a diffractometer to verify the structures of the synthesized products. The XRD patterns of the solid samples were collected in a D2 Physier diffractometer (D2 Phaser model; Brüker, Billerica, MA, USA) using CuKα1 radiation (2*θ* = 15–80). ^1^H and ^13^C NMR spectra were obtained with an AVANCE 500 MHz instrument at the Faculty of Chemistry (University of Gdańsk). The chemical shift values were determined in ppm (δ) and applied to tetramethylsilane used as a solvent (2.49 for ^1^H in DMSO*-d_6_*). ESI-MS spectra of Ru(III) complexes with STZ as well as SMZ were recorded in negative ion mode by directly injecting the complexes at a flow rate of 5 μL · min^−1^ using a Bruker Daltonics HCT Ultra high-resolution mass spectrometer equipped with a conventional ESI source. All measurements were performed at room temperature. Conductivity values were determined using an ELMETRON CC-401 conductivity meter, at 25 °C, in a water and methanol solvent mixture (*v*/*v* = 1:1), for all synthesized Ru(III) complexes, as well as the free form of sulfonamide ligands (STZ and SMZ), at a concentration of 1 mM. Thermal decomposition was examined using a TG209 Netzsch thermal equalizer. All experiments were carried out in an argon atmosphere. The analyzer was equipped with a programmed temperature controller that automatically maintains a constant temperature during thermal events. The TG weight loss was measured from 20 to 1100 °C at a heating rate of 15 °C·min^−1^. Electrochemical measurements were obtained using an Autolab PGSTAT204 potentiostat/galvanostat (Metrohm Autolab B.V., Utrecht, The Netherlands) which is controlled by Nova software. Steady-state fluorescence experiments were carried out at 25 °C using an FL 6500 spectrofluorometer (PerkinElmer, Waltham, MA, USA) equipped with a temperature controller and a 1.0 cm quartz multicell holder. Absorbance readings were obtained using an EnSpire microplate reader (PerkinElmer). All calculations were performed in OriginLab software.

### 3.3. Synthesis of [RuCl(OH_2_)(STZ)_2_]Cl_2_·H_2_O (1)

The Ru(III)-STZ complex was prepared by refluxing the 1:2 (Ru^III^: STZ) molar ratio of the mixture (1 mmole; 224 mg) of ruthenium(III) chloride monohydrate with sulfathiazole (2 mmole; 509 mg of STZ) in a freshly prepared anhydrous methanol. The reaction system was initially neutralized to a pH of about 8–9 and refluxed further by magnetic stirring for 3 h at a controlled, constant temperature of 45 °C. The resulting solution was left overnight to allow reduction (to approximately half of its volume). The complex product **(1)** was formed on the third day, and the obtained brown-colored micropowder was filtered off and washed with diethyl ether. The compound was dried for 2 days in a vacuum exsiccator in the presence of anhydrous CaCl_2_. The synthesis yield of compound **(1)** was estimated at about 77%. Elemental analysis of **(1)**; *calc.*: 28.67%C; 2.94%H, 11.15%N, 17.01%S: *found*: 28.78%C; 2.96%H; 11.14%N; 16.97%S. ATR signal positions *ṽ* [cm^−1^] and assignment: 458 (Ru-Cl); 1137 ν_s_(SO_2_); 1316 ν_as_(SO_2_); 3100 ν(NH); 1577 δ(NH); 3368 ν_s_(NH); 3470 ν_as_(NH); ^1^H NMR(DMSO-*d_6_*) δ [ppm]: 5.9 (s, 4H, NH_2_), 6.7–7.9 [m, 12H, Ar-H + SO_2_NH] [73], 8.3 (s, 2H, Ar-H); ^13^C NMR (125 MHz) δ [ppm]: 107.65, 108.71, 113.72, 128.52, 168.39; ESI/MS [m/z]: 753.6; [RuCl_3_C_18_H_22_O_6_S_4_N_6_+e^−^]^−^; XRD powder signal positions [2*Θ*]: 6.62561; 6.28820; total number of Cl^−^ potentiometrically established in **(1)** formula: 3; number of Cl^−^ as counter ions (outside coordination sphere): 2 (Figure S22).

### 3.4. Preparation of the [RuCl_2_(SMZ)_2_]Cl Complex (2)

The Ru(III)-SMZ complex was prepared by refluxing 1:2 (Ru^III^: SMZ) molar ratio of the mixture (1 mmole; 225 mg) of ruthenium(III) chloride monohydrate with sulfamerazine (2 mmole; 528 mg of SMZ) in a non-aqueous (99.99%) ethanol. The reaction system was heated and stirred for 2 h at 60 °C, with the apparatus protected using a drying agent (CaCl_2_). The resulting colored solution was filtered off and allowed to evaporate slowly. The crude product was formed on the next day. The deep-violet-colored precipitate was filtered off and washed with diethyl ether. The solid sample of **(2)** was used after exactly 5 days of drying in a vacuum exsiccator over anhydrous CaCl_2_. The synthesis yield of compound **(2)** was estimated at 64%. Elemental analysis of **(2)**; *calc.:* 35.90%C; 3.29%H, 15.22%N, 8.72%S: *found:* 35.87%C; 3.30%H; 15.24%N; 8.70%S; ATR signal positions *ṽ* [cm^−1^] and assignments: 448 (Ru-Cl); 1156 ν_s_(SO_2_); 1302 ν_as_(SO_2_); 3251 ν(NH); 1633 (δ NH); 3363 ν_s_(NH); 3466 ν_as_(NH); ^1^H NMR(DMSO-*d_6_*) δ [ppm]: 3.45 (d, 6H CH_3_), 5.8 (s, 4H, NH_2_), 6.7–7.9 [m, 12H, Ar-H + SO_2_NH], 8.3 (s, 4H, Ar-H); ^13^C NMR (125 MHz) δ [ppm]: 112.50, 115.23, 125.48, 130.51, 153.34, 158.1, 168.40; ESI/MS [m/z]: 735.7; [RuCl_3_C_22_H_24_O_4_S_2_N_8_+e^−^]^−^; XRD powder signal positions [2*Θ*]: 19.6105; 22.37689; 29.47013; 32.58513; total number of Cl^−^ potentiometrically established in **(2)** formula: 3; number of Cl^−^ as counter ions (outside coordination sphere): 1 (Figure S22).

### 3.5. Interaction Affinity to CT-DNA Biomolecule

UV-Vis titration with *CT*-DNA assay was carried out in Tris-HCl buffer solution (5 mM Tris-HCl, 50 mM NaCl, pH 7.40). Absorption measurements were obtained using a Thermo Scientific Evolution 300 double-beam spectrophotometer equipped with a Lauda temperature programmer (E100; 25 °C). Quartz cuvettes (StarnaGmbH; Pfungstadt, Germany, 1/GL14/C) with a path length of 10 mm and a scanning rate of 240 nm·min^−1^ were used to register intermolecular interaction processes. UV-Vis spectra of the tested compounds—STZ, SMZ, **(1),** and **(2)**—were obtained at wavelengths of 200–650 nm in the Tris-HCl buffer (pH 7.40; 5 mM/50 mM NaClO_4_). The absorption spectra were recorded after each addition of constant (identical in volume) amounts of *CT*-DNA Tris-solution. The concentrations were established independently in preliminary studies performed individually for each studied object, to obtain appropriate results for specific sulfa objects carefully selected for the work. For a solution of *CT*-DNA in Tris-HCl, a UV-Vis absorbance value of 1.81 was determined at 260 nm—a standard value indicating that DNA is sufficiently free of protein. Titrations were done automatically by using a CerkoLab microinjector at a constant temperature of 25 °C in the range of 200–400 nm. The concentration of freshly prepared *CT*-DNA was calculated based on the absorbance value at 260 nm as well as the calibration curve [ε_DNA_ 6600 (base pairs) M^−1^·cm^−1^] [74]. STZ, SMZ, and complexes **(1)** or **(2)** were dissolved in Tris-HCl buffer to yield samples of 10 mL solutions, each with a mass concentration of 14, 66, 28, and 80 μM, respectively. A 1.5 mL solution with the appropriate titrand was placed in a quartz cuvette and consequently titrated by successive addition of a 190 μM solution of *CT-*DNA at specific and constant time intervals (45 s) to allow for interactions between the components of the system. The concentration of hybridized and etched *CT*-DNA (titrant) was determined based on the calibration curve obtained at 260 nm in the preliminary research (Figure S23). The concentration of *CT*-DNA biomolecule was maintained the same for STZ, SMZ, **(1),** and **(2)**.

### 3.6. Fluorescence Spectroscopy

The fluorescence emission spectra (λ_ex_ = 260 nm) of all the studied compounds were recorded from 300 to 700 nm both in the absence and presence of *CT*-DNA at increasing concentrations (starting from 1.87 mM in Tris-HCl, pH = 7.4). The typical concentrations of the studied compounds were in the range of 0.018–0.146 mM. Solutions were stored in the dark to minimize undesired photoreactions between measurements. In the fluorescence titration experiments, 1.5 mL solution containing the analyte (SMZ, Ru(III)-SMZ, STZ, Ru(III)-STZ, respectively) was titrated with a successively added stock solution of *CT*-DNA in a microinjector. The solutions were allowed to stand for 5 min to equilibrate. Based on the intensity of the band at 570 nm (corresponding to the maximum emission of all compounds), the values of association constants (K_A_) and other parameters were calculated.

### 3.7. Electrochemical Profile

Measurements were obtained in a single-compartment, three-electrode cell by performing cyclic voltammetry (CV) and differential pulse voltammetry (DPV) techniques. The working electrode used was a 2 mm glassy carbon (GC) electrode. It was carefully polished before each experiment using a 0.5 μM alumina suspension (Buehler). A platinum wire was used as an auxiliary electrode. All potentials were measured vs. a silver/silver chloride (Ag/AgCl) reference electrode, wherein a double-junction electrode with aqueous sodium chloride (1 M NaCl) solution in 0.1 M TBAP in methanol was used as aprotic media. The compounds used for obtaining voltammetric measurements were in the concentration range of 10^−3^–10^−4^ M and were dissolved in 0.1 M TBAP in DMSO or 0.1 M sodium perchlorate in water or Tris-HCl buffer (pH 7.4). All voltammetric measurements were performed at a temperature of 298 K, and the solutions were degassed by passing argon. At the beginning of the experiment, the cell was held at the start potential for 10 s of silence time. To ensure the reproducibility of results, the voltammograms were recorded several times for each experiment. The obtained data were processed in *OriginLab* software.

### 3.8. MIC Determination

The minimal inhibitory concentration (MIC) or the lowest concentration that inhibits bacterial growth was determined for the solutions of STZ, [RuCl(OH_2_)(STZ)_2_]Cl_2_ · H_2_O **(1)**, SMZ, [RuCl_2_(SMZ)_2_]Cl **(2)**, and RuCl_3_ · H_2_O, freshly prepared in DMSO (at a concentration of 15 mg · mL^−1^) on the day of the assay. The STZ, Ru(III)-STZ complex, SMZ, Ru(III)-SMZ complex, and RuCl_3_·H_2_O were assayed at a concentration range of 2–512 μg·mL^−1^ while the concentration of ciprofloxacin (control) used was 0.064–256 μg·mL^−1^. The MIC analysis was performed using the broth macro dilution method with Mueller-Hinton II broth. An initial inoculum of 5 · 10^5^ colony-forming unit (cfu)·mL^−1^ was used for the analysis [75]. The incubation was performed for 18 h at 37 °C. All experiments were performed in triplicate.

### 3.9. Cytotoxicity Assay

To determine the cytotoxicity of STZ and SMZ free ligands, as well as complexes (1) and (2) the WST-1 assay, was performed. The human epidermal keratinocytes (HaCaT) were grown in DMEM (with a high concentration of glucose and sodium pyruvate), supplemented with 10% FBS and antibiotics (streptomycin and penicillin) at a concentration of 100 U·mL^−1^. The human prostate cancer cell line (PC3) was grown in F-12K medium supplemented with 10% FBS and antibiotics (streptomycin and penicillin) at the same concentration. The human breast cancer cells (MCF-7) were grown in RPMI medium supplemented in the same way as DMEM and F-12K medium. The HaCaT, PC3, and MCF-7 cells were seeded into a 96-well plate at a density of 4000 cells per well and incubated (37 °C and 5% CO_2_) overnight. After incubation, the medium was replaced with a fresh one and the tested compounds were added to the plate at different concentrations: 0 µM for the control; 50, 110, 170, and 200 µM for the complexes; and 200 µM for the ligands. The treated cells were incubated (37 °C and 5% CO_2_) for 24 and 48 h, and then, 10 μL of WST-1 salt aqueous solution was added to each well and incubated again for 4 h. The absorbance was measured at 440 nm (reference: 660 nm). The vitality of control was taken as 100%. The obtained results were analyzed in GraphPad Prism software (San Diego, CA, USA). Statistical significance was evaluated using a one-way analysis of variance (ANOVA) followed by Dunnett’s multiple comparison test. Data were obtained from three experiments, and each treatment condition was assayed in triplicate. The differences were considered significant at *p* < 0.05.

## 4. Conclusions

Antibiotic resistance has become a global problem in recent years. Literature data show that metal-based drugs may be a viable solution to overcome this issue. In this study, a wide range of techniques was used to thoroughly characterize the newly synthesized [RuCl(OH_2_)(STZ)_2_]Cl_2_ · H_2_O **(1)** and [RuCl_2_(SMZ)_2_]Cl **(2)** complexes. Moreover, the same experiments have been carried out for free ligands STZ and SMZ, allowing for comparative analysis. Taking into account that Ru(III) complex compounds are usually octahedral, as indicated by their empirical formulas and characterization, it is reasonable to suggest the asymmetric coordination of both Ru(III) sulfonamide compounds **(1)** and **(2)**, which were synthesized.

The affinity of the studied compounds, described by the values of binding constants, was determined by two independent techniques: spectrophotometry and spectrofluorimetry. The results obtained from experiments proved that all the examined compounds interacted with *CT*-DNA. The electrochemical profile confirmed, as expected, that both Ru(III) complexes were reduced to their Ru(II) analogs, which is very important for their potential anticancer application. The WST-1 assay showed that Ru(III)-STZ and Ru(III)-SMZ were characterized by higher cytotoxicity compared to the free ligands. For example, in the MCF-7 line treated with Ru(III)-STZ complex, the increase in anticancer activity was equal to 45% compared to the activity of the free ligand STZ. The results of microbial experiments also indicated that both synthesized complexes exhibited better antimicrobial properties compared to the free ligands. This fact confirms that metallodrugs can be a promising alternative to traditional antibiotic therapy. Moreover, the data obtained from biological experiments indicated the need for further research aimed at identifying the mechanism of action of the synthesized complexes—such research is already in progress in our group and will be the subject of our further papers. The approach described in this article will allow the rational design of new derivatives with better properties.

To summarize, the results presented here may enhance the understanding of the coordination compounds of Ru(III) ions and sulfonamides, paving the way for further research. Moreover, the data obtained in the study confirm the thesis that the complexation of compounds with metal ions may result in complexes with improved properties.

## Figures and Tables

**Table 1:** Selected ATR spectral data of substrates and synthesized Ru(III) coordination products together with the signal assignments indicate the donor atoms of sulfonamides involved in binding the ionic metallocenter. All values of table are in [cm^−1^].

		Sulfonamido (–SO_2_NH–)			Anilino (–NH_2_)		
	ν(Ru-Cl)	ν_s_(SO_2_)	ν_as_(SO_2_)	ν(NH)	δ(NH)	ν_s_(NH)	ν_as_(NH)
RuCl_3_·H_2_O	458	-	-	-	-	-	-
Sulfathiazole STZ	-	1129	1318	3271	1590	3319	3351
[RuCl(OH_2_)(STZ)_2_]Cl_2_·H_2_O (1)	458	1137	1316	3100	1577	3368	3470
Sulfamerazine SMZ	-	1156	1324	3251	1633	3375	3484
[RuCl_2_(SMZ)_2_]Cl (2)	448 *	1156	1302	3251	1633	3363	3466

ν_as_ = stretching (antisymmetric); ν_s_ = stretching (symmetric); δ = bending (scissoring); * = doubled intensity of band compared to signal intensity of **(1)**.

**Table 2:** Conductivity of solutions in the mixture H_2_O:MeOH (*v*/*v* = 1:1; 1 mM) together with electronic data of complexes’ studied samples (DMSO).

Compound Formula (Geometry)	Conductivity (μS)	UV-Vis Bands (cm^−1^)	Assignments
[RuCl(OH_2_)(STZ)_2_]Cl_2_·H_2_O **(1)** (bipyramidal tetragonal)	127.7	38,314	Intraligand charge transfer
		34,247	^2^*T*_2g_ → ^2^*A*_2g_, ^2^*T*_1g_
		22,988	^2^*T*_2g_ → ^2^*E*_g_
		19,342	^2^*T*_2g_ → ^4^*T*_1g_
[RuCl_2_(SMZ)_2_]Cl **(2)** (octahedral distorted)	12.58	41,322	Intraligand charge transfer
		39,215	^2^*T*_2g_ → ^2^*A*_2g_, ^2^*T*_1g_
		28,248	^2^*T*_2g_ → ^2^*E*_g_
		18,797	^2^*T*_2g_ → ^4^*T*_1g_

**Table 3:** The comparative analysis of thermal decomposition data of the sulfa drugs and their Ru(III) complexes.

Compound	Amount (mg)	Steps	Temp. Range (°C)	DTG (+) ENDO (−) EGZO	Weight Loss (%)		Decomposed Fragment Assignments
STZ					*Calc.*	*Found*	
	9.712	1st	200–300	225 (+)	18.01	18.45	CO + H_2_O
		2nd	300–440	335 (+)	12.53	12.52	C_2_H_2_ fragment
		3rd	440–1090	500 (+)	42.69	42.13	C_3_H_4_SN_2_ fragment + ½ H_2_O
		Residue				26.90	* NE
[RuCl(OH_2_)(STZ)_2_]Cl_2_ ·H_2_O (1)					*Calc.*	*Found*	
	12.375	1st	19–170	98 (+)	4.77	4.22	H_2_O (outside)
		2nd	170–580	250 (+)	36.29	35.59	Water molecule + one ligand STZ decompostion to carbon and nitrogen oxides
		3rd		300 (+)			
		4th		375 (+)			
		5th	580–1090	750 (+)	31.00	30.09	Cl_2_ + second STZ ligand fragment C_7_H_5_N_3_O_2_ decomposed to carbon, nitrogen oxides, and water
		6th		887 (+)			
		Residual mass				30.10	RuS_2_ and residual carbons
SMZ					*Calc.*	*Found*	
	7.653	1st	200–300	280 (+)	51.45	51.39	C_3_H_5_NS fragment of 1,3-diazine
		2nd	300–325	310 (+)	7.18	7.47	NH_3_ + H_2_ (reductor)
		3rd	325–800	350 (+)	14.75	14.92	CO + ½ H_2_O
		Residue				26.22	* NE
[RuCl_2_(SMZ)_2_]Cl (2)					*Calc.*	*Found*	
	10.328	1st	190–250	225 (+)	3.66	3.57	-HCl
		2nd	250–370	275 (+)	48.70	47.38	C_11_H_12_N_4_O_2_S decomposition of *SMZ* + C_5_N_2_H_6_ (4-methyl-1,3-diazine)
		3rd	370–450	380 (+)	23.37	24.04	C_6_H_8_N_2_SO_2_ (*SMZ* defragmentation)
		Residual mass				25.01	RuO_2_ and residual carbons

* NE—not established during the measurement.

**Table 4:** Summary of the binding data obtained from the titration of fluorescent probes *CT*-DNA (K_sv_—dynamic quenching constant (the Stern–Volmer constant), K_D_—dissociation constant, K_A_—association constant, n—number of the binding site).

	[RuCl_2_(SMZ)_2_]Cl		[RuCl(OH_2_)(STZ)_2_]Cl_2_	
		R^2^		R^2^
K_SV_ (570)	75790 ± 690	0.999	77651 ± 1185	0.998
K_D_ (570)	1.24·10^−5^ ± 5.89·10^−8^	0.999	1.34·10^−5^ ± 1.60·10^−7^	0.999
log K_A_ (570)	4.67 ± 0.05	0.999	5.25 ± 0.06	0.999
K_A_ (570)	46773	0.999	179420	0.999
n (570)	0.954 ± 0.01	0.999	1.08 ± 0.01	0.999
	**SMZ**		**STZ**	
		**R^2^**		**R^2^**
K_SV_ (570)	80583 ± 827	0.999	66952 ± 267	0.999
K_D_ (570)	1.34·10^−5^ ± 1.14·10^−7^	0.999	1.17·10^−5^ ± 9.25·10^−8^	0.999
log K_A_ (570)	5.10 ± 0.02	0.999	4.48 ± 0.02	0.999
K_A_ (570)	127544	0.999	30477	0.999
n (570)	1.048 ± 0.00	0.999	0.93 ± 0.00	0.999

**Table 5:** Values of cathodic potentials E_c_ of studied compounds in DMSO recorded using CV and DPV and values of the diffusion coefficient D.

Compound	E_c_ [V] (CV)	E_c_ [V] (DPV)	E_c_ [V] (CV, 25 mV·s^−1^)	E_c_ [V] (CV, 225 mV·s^−1^)	D (cm^2^ ·s^−1^)
STZ	−2.377	−2.325	−2.401	−2.477	1.157·10^−6^
[RuCl(OH_2_)(STZ)_2_]Cl_2_·H_2_O (1)	−2.202	−1.906	−2.190	−2.328	3.959·10^−7^
SMZ	−1.689	−1.597	−1.658	−1.701	5.916·10^−8^
[RuCl_2_(SMZ)_2_]Cl (2)	−1.677	−1.582	−1.664	−1.714	9.408·10^−8^

**Table 6:** Comparison of minimal inhibitory concentrations (MIC) of STZ, [RuCl(OH_2_)(STZ)_2_]Cl_2_·H_2_O **(1)**, SMZ, [RuCl_2_(SMZ)_2_]Cl **(2)** and RuCl_3_ · H_2_O and ciprofloxacin.

	MIC (Minimal Inhibitory Concentrations) (µM)			
	*Staphylococcus aureus* ATCC 25923	*Enterococcus faecalis* ATCC 19433	*Escherichia coli* ATCC 25922	*Pseudomonas aeruginosa* ATCC 27853
	Gram-Positive		Gram-Negative	
STZ	501	63	251	1002
[RuCl(OH_2_)(STZ)_2_]Cl_2_ ·H_2_O **(1)**	340	42	169	679
SMZ	>1930	121	242	>1930
[RuCl_2_(SMZ)_2_]Cl **(2)**	>696	87	174	>696
RuCl_3_·H_2_O	>2270	>2270	>2270	>2270
Ciprofloxacin *	1.500	1.500	<0.181	3.000

* Control.

## Supplementary Materials

The following are available online at https://www.mdpi.com/article/10.3390/ijms222413482/s1.
