# Supplementary material for: Stimulation of Sulfonamides Antibacterial Drugs Activity as a Result of Complexation with Ru(III): Physicochemical and Biological Study
Source: Int J Mol Sci. 2021 Dec 15;22(24):13482. doi: 10.3390/ijms222413482 (PMC8708937; doi:10.3390/ijms222413482)
Supplement: Supplementary file 1 [file ijms-22-13482-s001.zip › ijms-1482249-supplementary.pdf]

International Journal of
Molecular Sciences

Supplementary materials

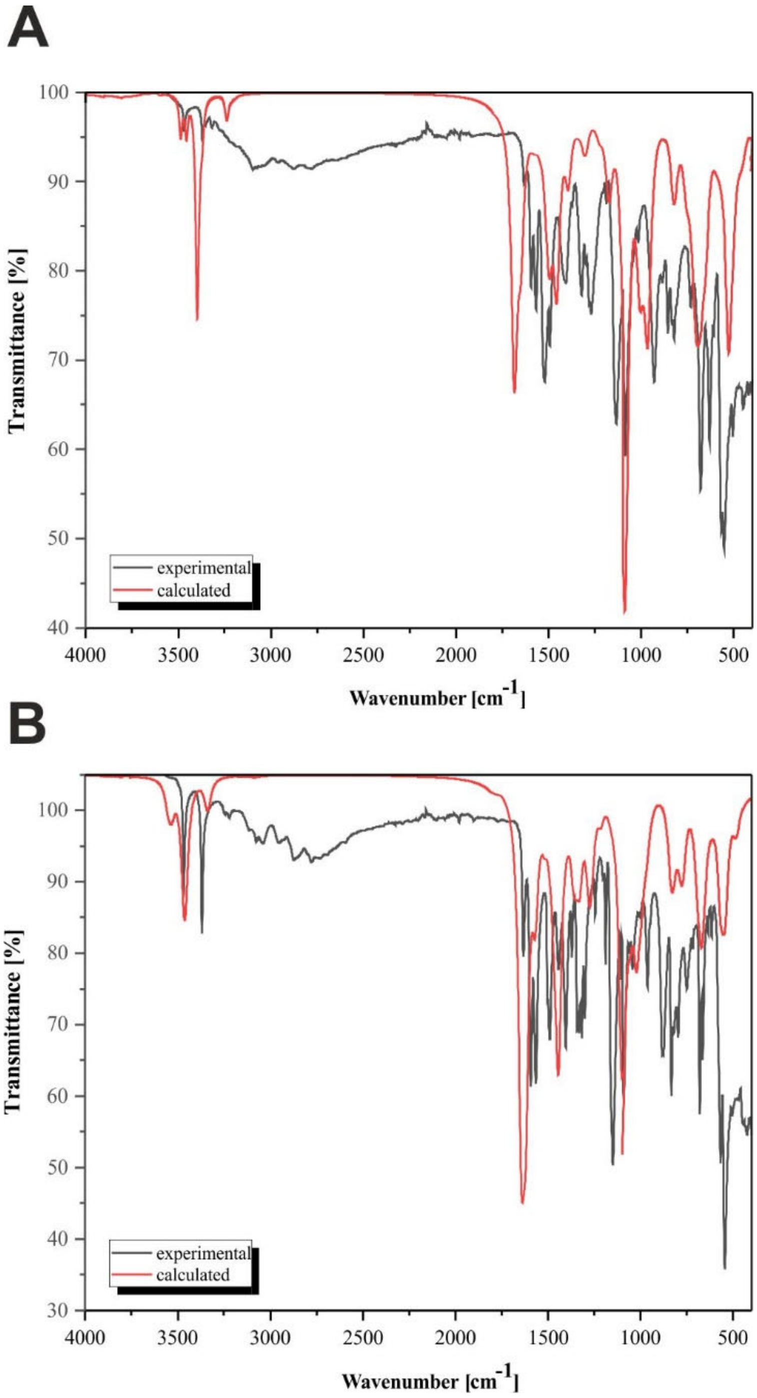

Figure S1. Experimental (ATR) and theoretical (IR)* signals for (**A**) $[RuCl(OH_2)(STZ)_2]Cl_2 \cdot H_2O$, and (**B**) $[RuCl_2(SMZ)_2]Cl_2$. *All calculations were performed with the GAUSSIAN16 (Rev.C.01) package.

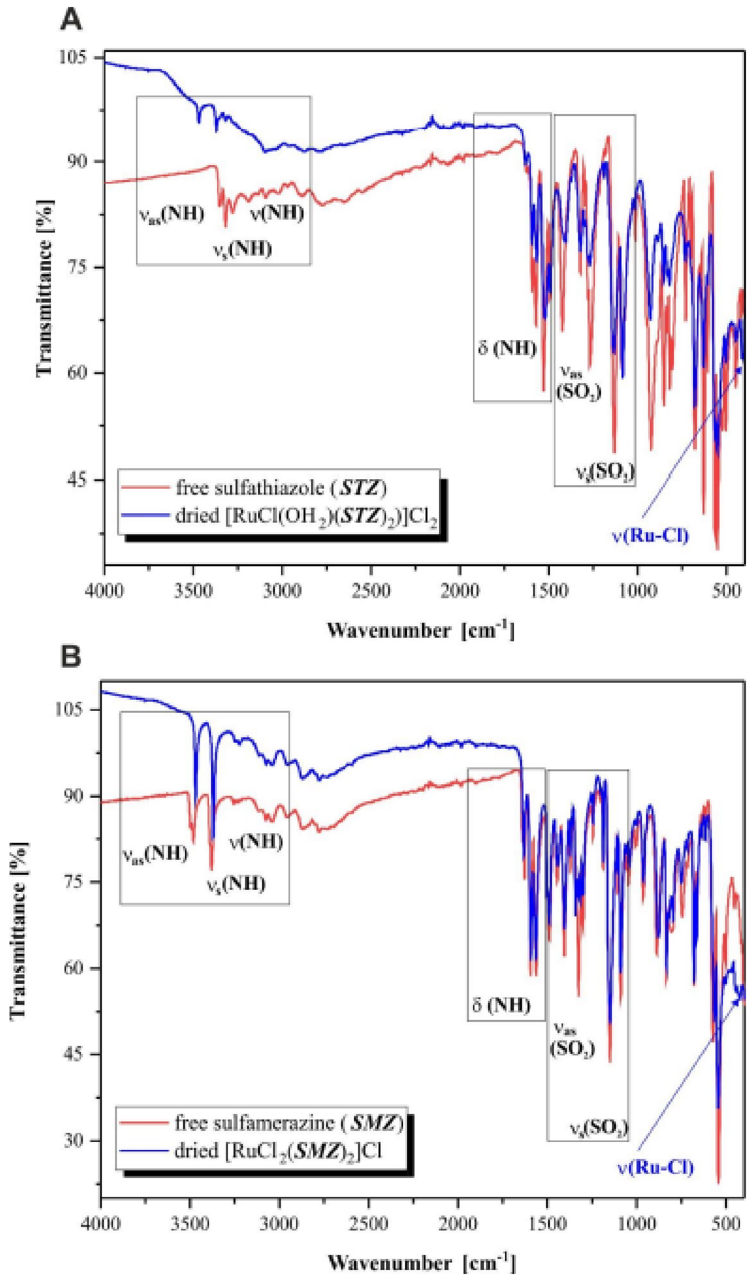

Figure S2. Oscillatory spectra (ATR) of both Ru(III) complexes studied **(1)** (**A**) $[RuCl(OH_2)(STZ)_2]Cl_2 \cdot H_2O$, and **(2)** (**B**) $[RuCl_2(SMZ)_2]Cl_2$ together with free ligands STZ, SMZ, collected respectively.

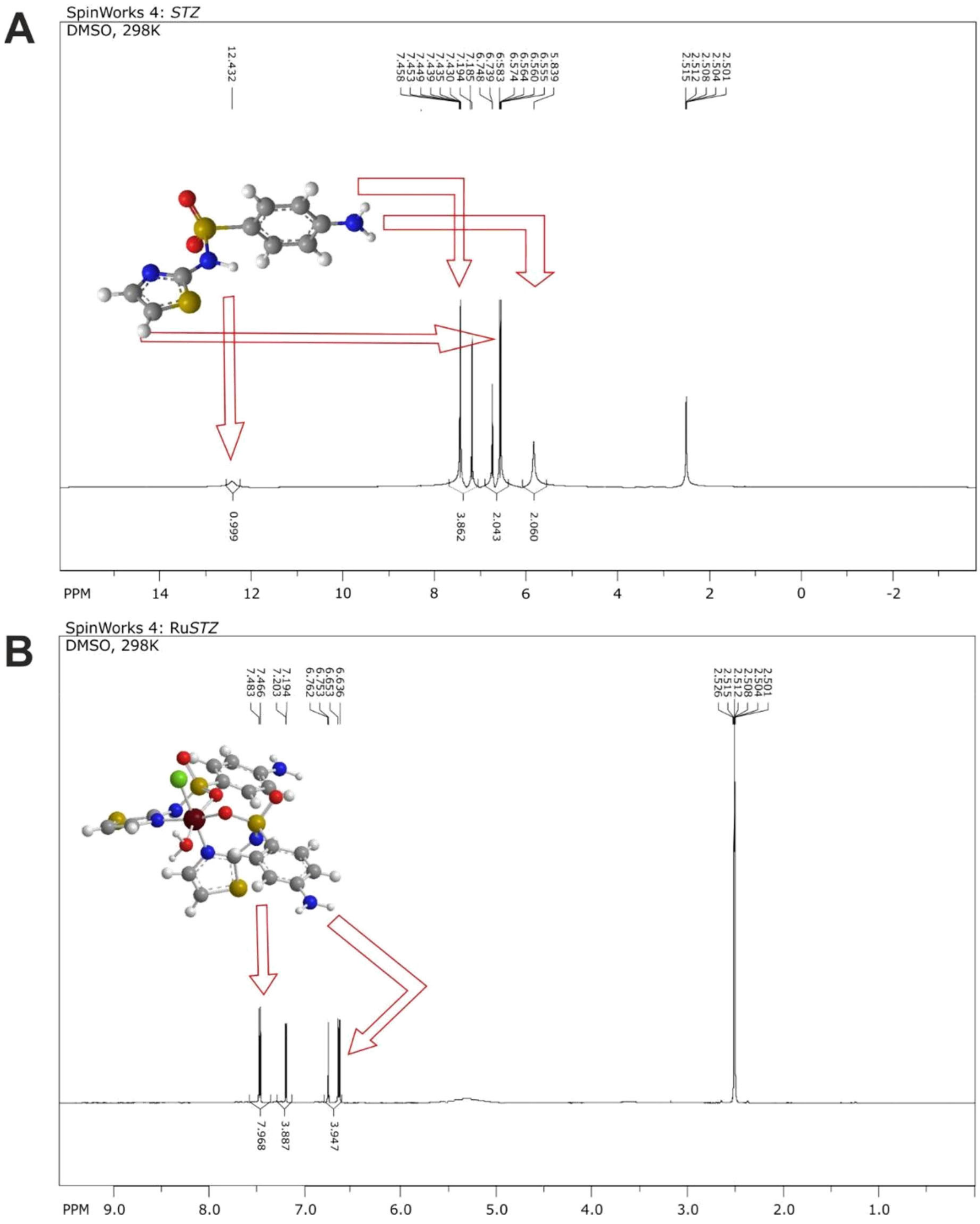

Figure S3. ^{1}H NMR spectra of the compounds studied: (**A**) STZ, (**B**) Ru(III)-STZ complex **(1),** measured in DMSO-d6 at concentrations 10 mM and temperature 25 °C.

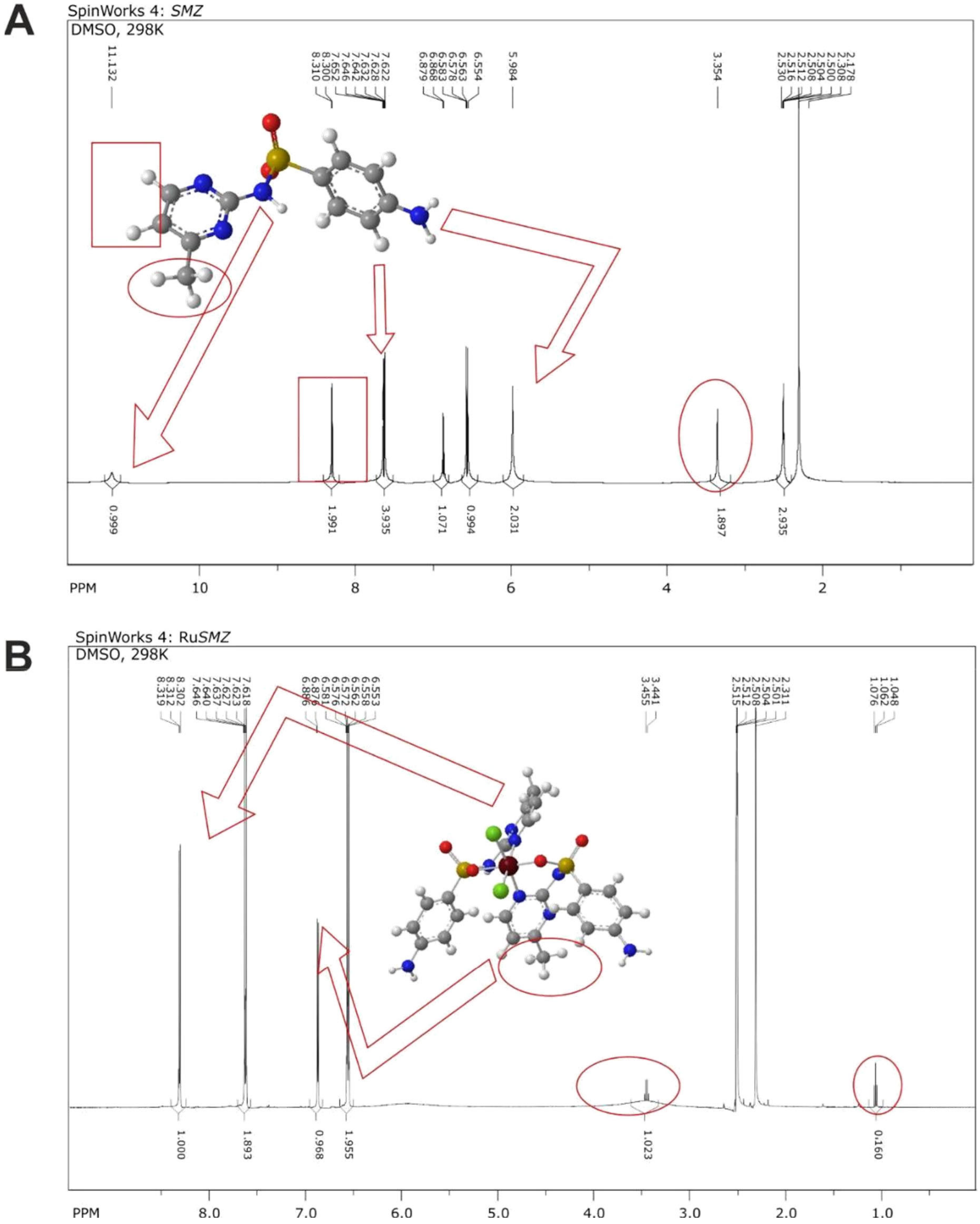

Figure S4. ^{1}H NMR spectra of the compounds studied: (**A**) SMZ, and (**B**) Ru(III)-SMZ complex. **(2)** measured in DMSO-d6 at concentrations 10 mM and temperature 25°C.

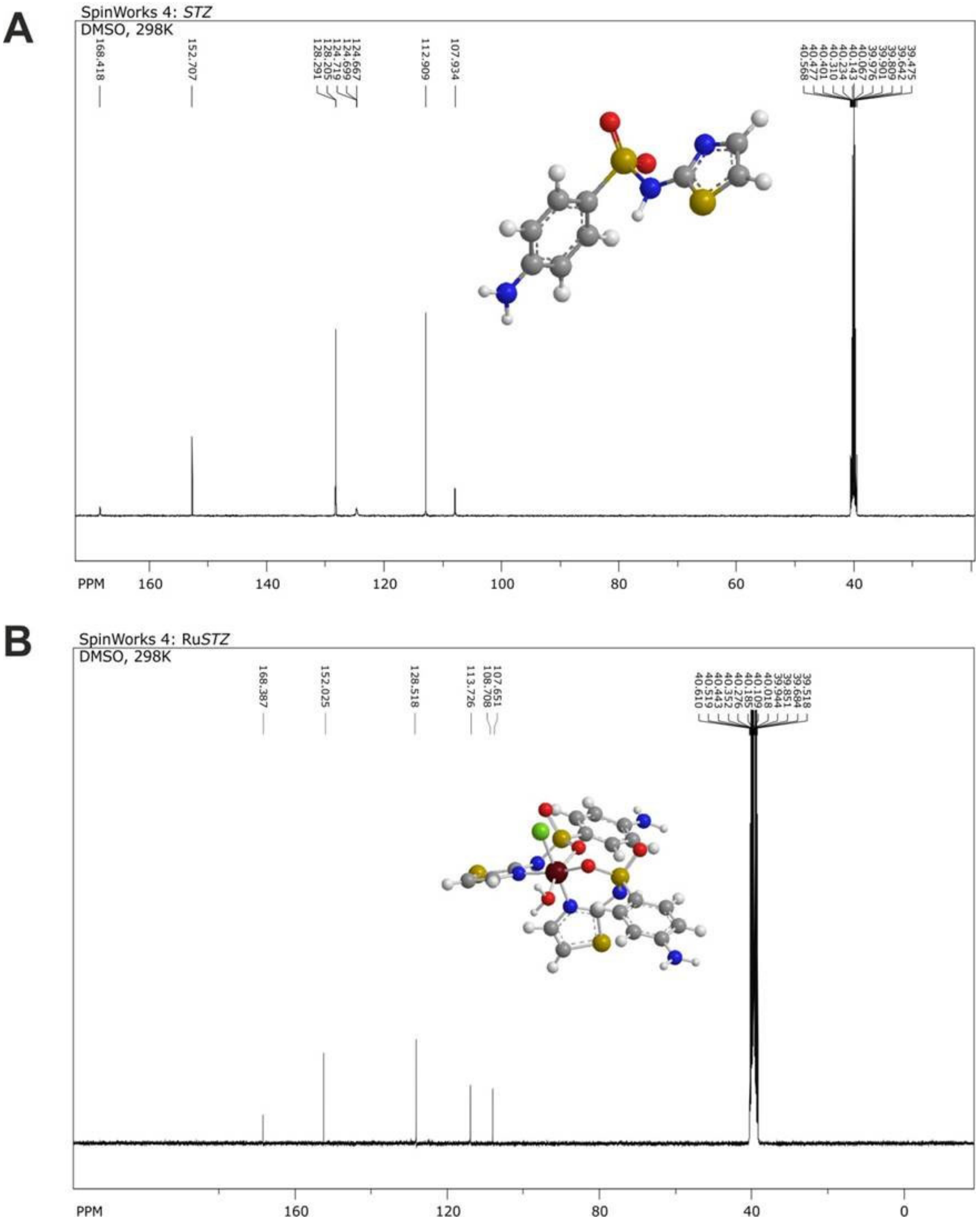

Figure S5. ^{13}C NMR spectra of compounds studied: (**A**) STZ, (B) Ru(III)-STZ complex **(1),** measured in DMSO-d_6 at concentrations 10 mM and temperature 25 °C.

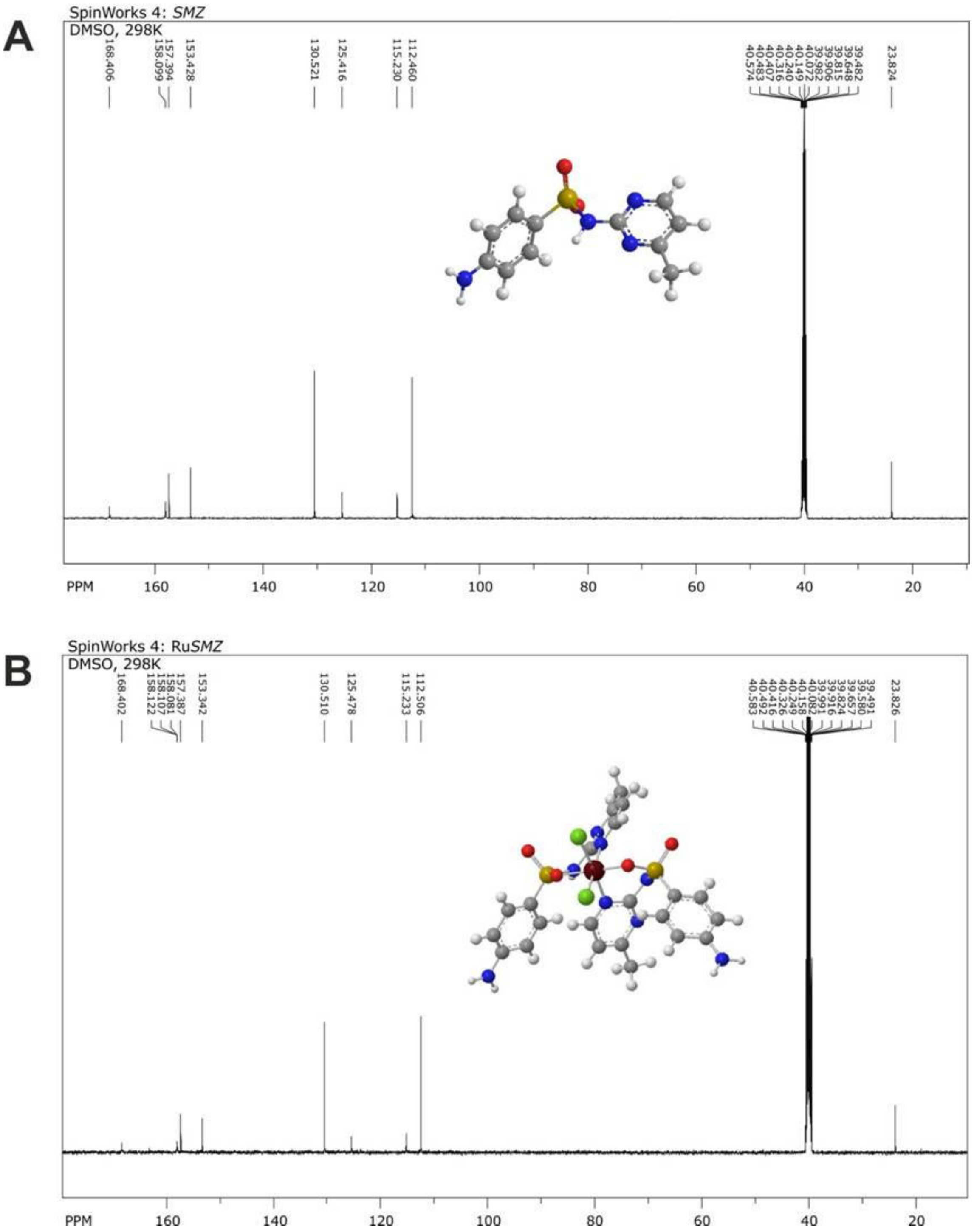

Figure S6. ^{13}C NMR spectra of compounds studied: (**A**) SMZ and (**B**) Ru(III)-SMZ complex **(2)** measured in DMSO-d_6 at concentrations 10 mM and temperature 25°C.

Table S1. XRD diffractogram data obtained for the synthesized complexes.

**Peaks Position, 2 Theta (degrees)**	**FWHM (degrees)**	**D (nm)**	**D Average (nm)**
	**$[RuCl(OH_2)(STZ)_2]Cl_2 \cdot H_2O$ (1)**		

23.11906<br>45.48605	6.62561<br>6.28820	1.223851<br>1.369875	1.30
	$[RuCl_2(SMZ)_2]Cl$ **(2)**		
**Peaks Position, 2 Theta (degrees)**	**FWHM (degrees)**	**D (nm)**	**D Average (nm)**
19.61050<br>22.37689<br>29.47013<br>32.58513	0.31927<br>0.31231<br>0.22484<br>0.28555	25.25153<br>25.93001<br>36.53462<br>28.98500	29.18

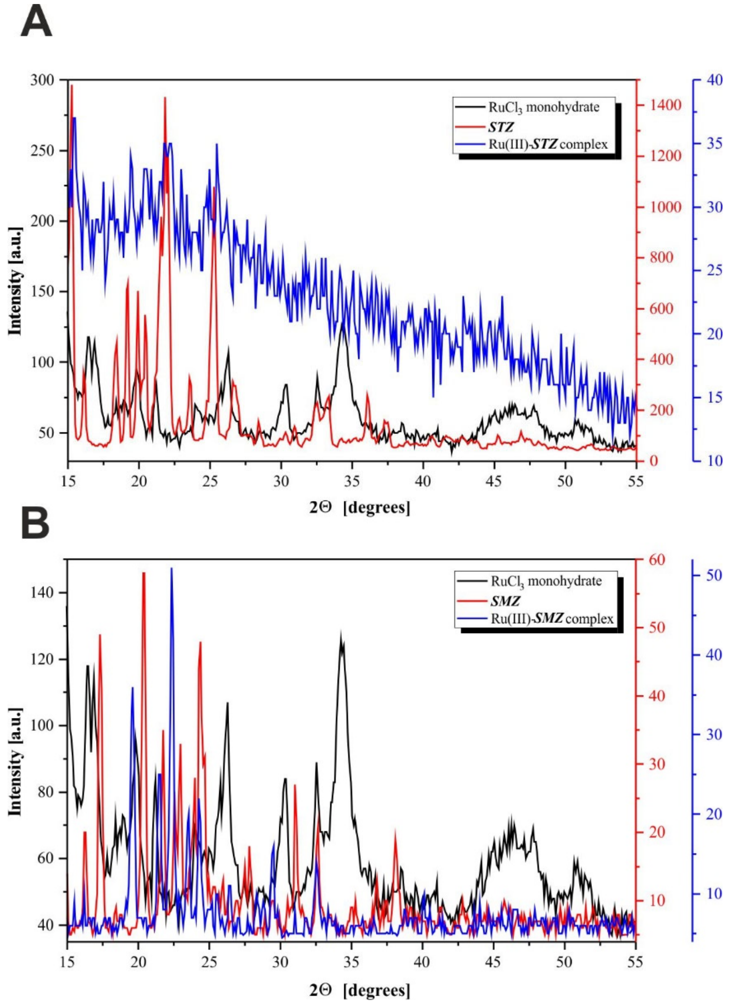

Figure S7. XRD diagrams compilation obtained for both synthetic systems studied: (**A**) spectra of substrates, $RuCl_3$ monohydrate pure sulfamerazine ligand compared with the complex **(1)** diffractogram; (**B**) spectra of substrates, $RuCl_3$ monohydrate and sulfathiazole collected together with the complex **(2)** diffractogram.

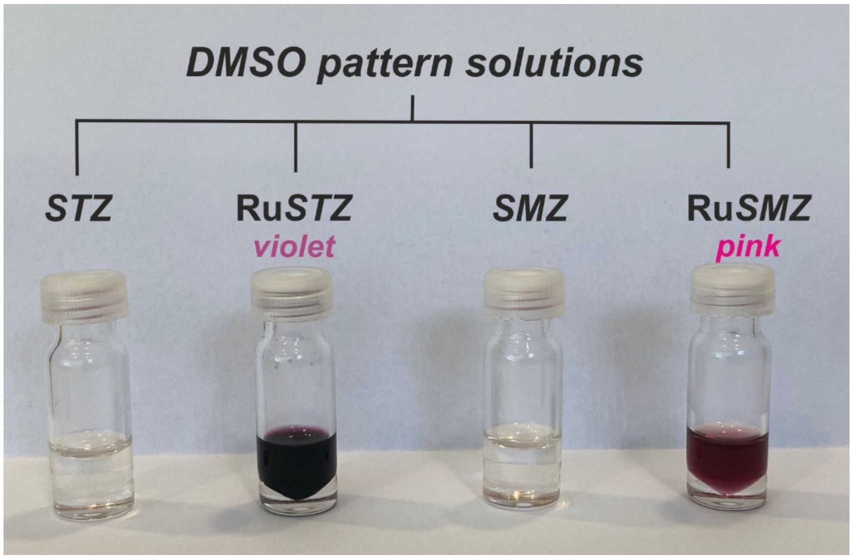

Figure S8. Photos of STZ, SMZ as well as $[RuCl(OH_2)(STZ)_2]Cl_2$, $[RuCl_2(SMZ)_2]Cl$ solutions studied and used during UV-Vis analyses.

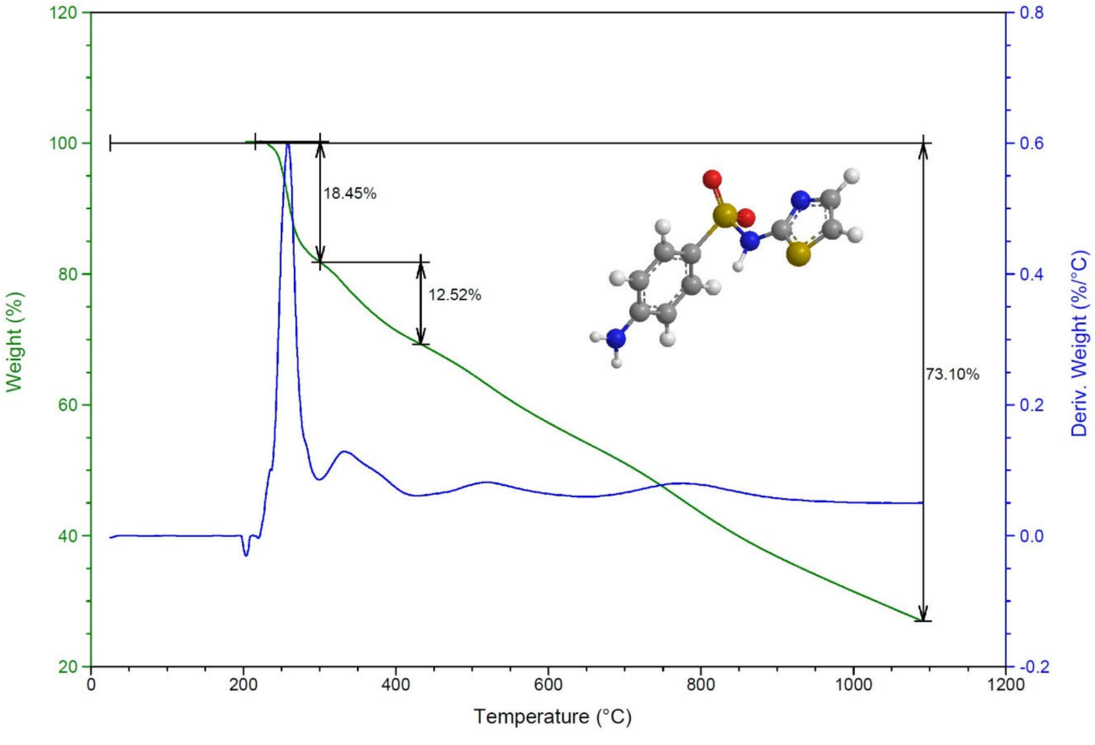

Figure S9. TG-DTG curves of sulfathiazole (STZ).

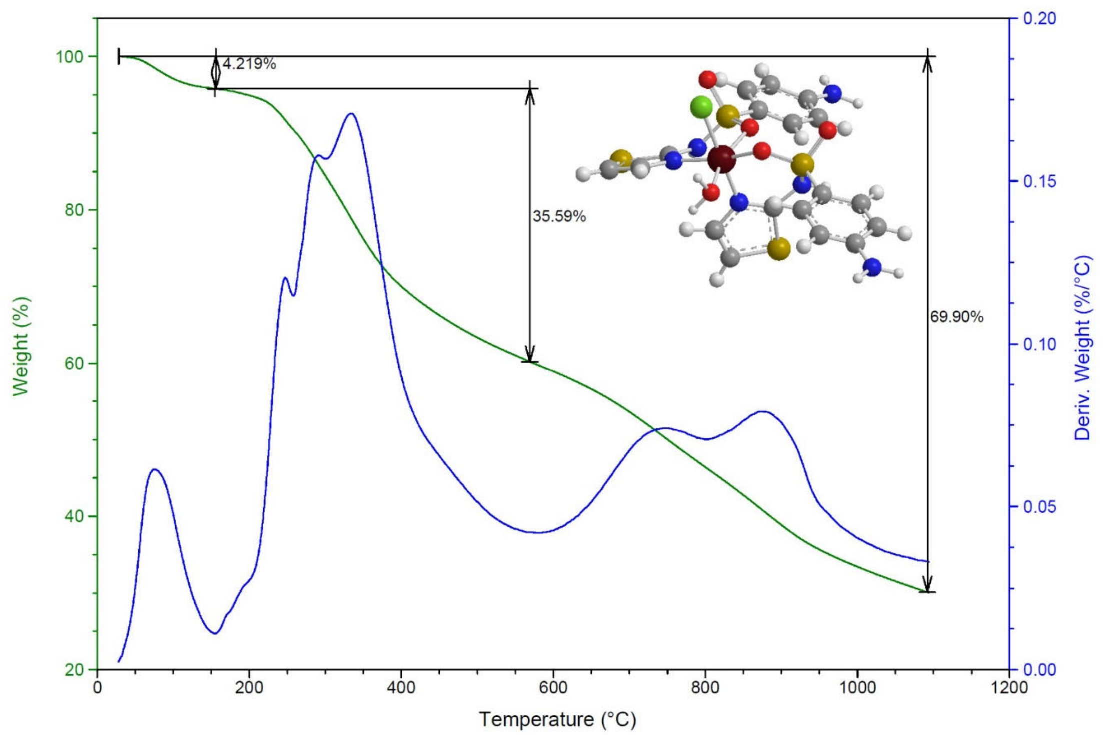

Figure S10. TG-DTG curves of Ru(III) complex with STZ.

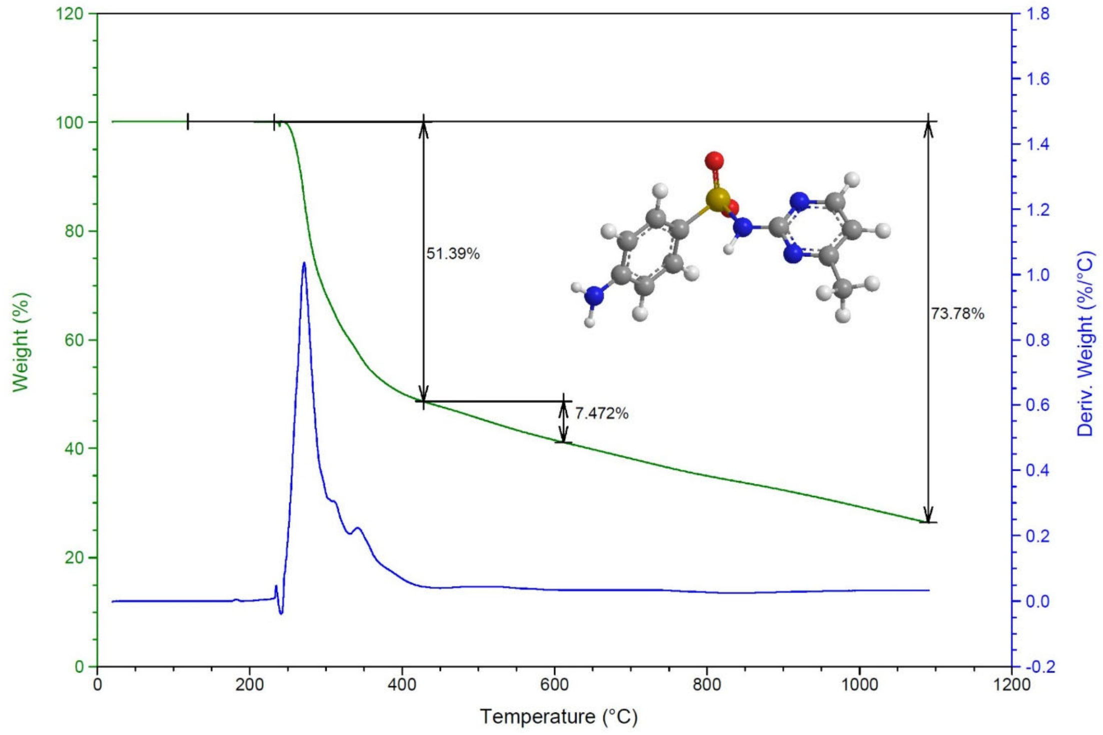

Figure S11. TG-DTG curves of sulfamerazine (*SMZ*).

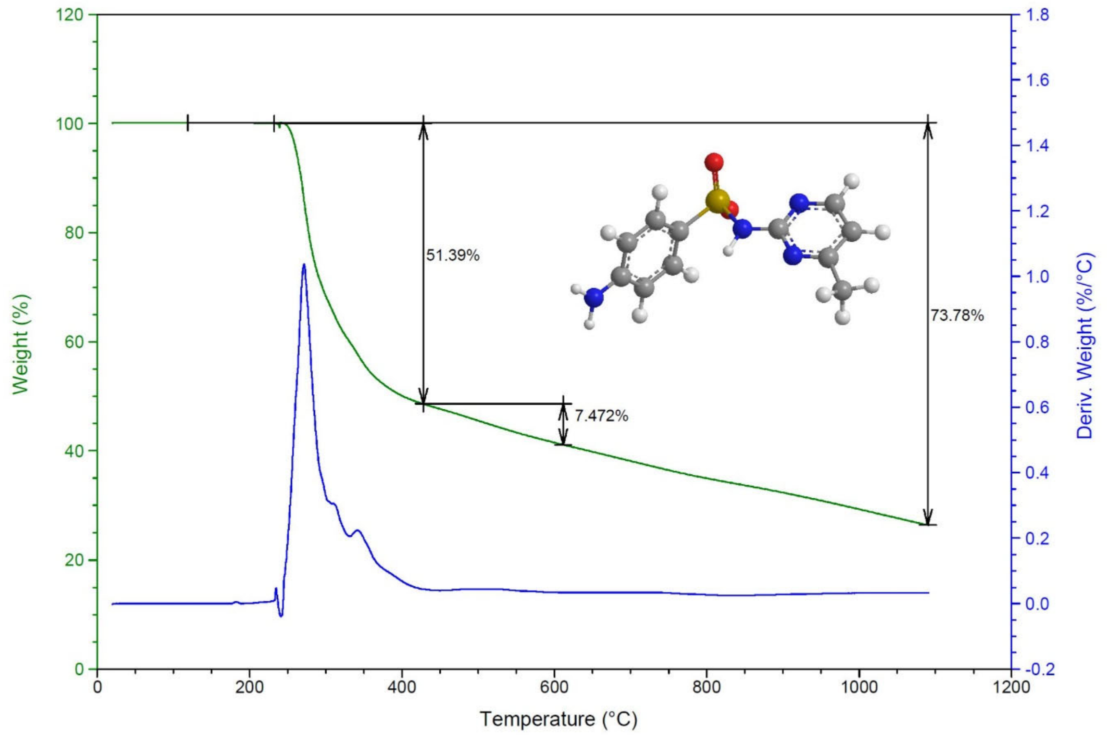

Figure S12. TG-DTG curves of Ru(III) complex with SMZ.

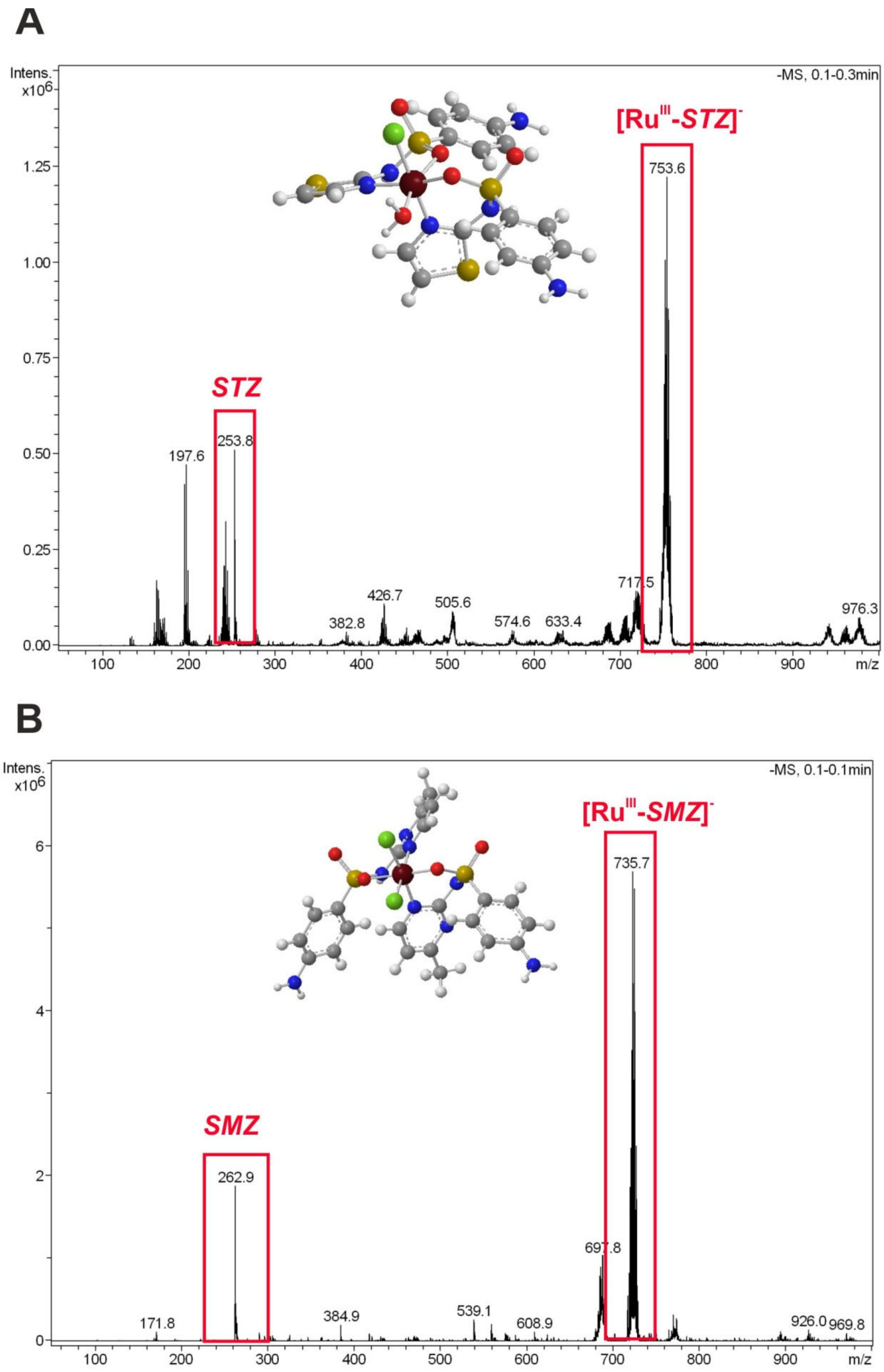

Figure S13. ESI-MS spectra of complexes: (**A**) $[RuCl(OH_2)(STZ)_2]Cl_2{\cdot}H_2O$; (**B**) $[RuCl_2(SMZ)_2]Cl$.

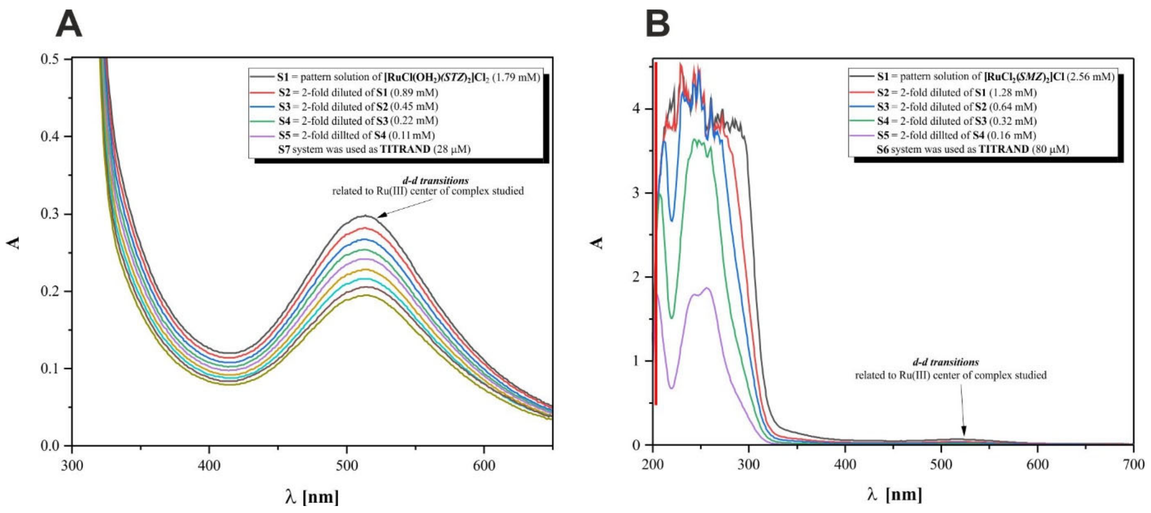

Figure S14. Electronic spectra (UV-Vis) of both coordination compounds with different concentrations (**A**) $[RuCl(OH_2)(STZ)_2]Cl_2$, (**B**) $[RuCl_2(SMZ)_2]Cl$ (the preliminary investigation).

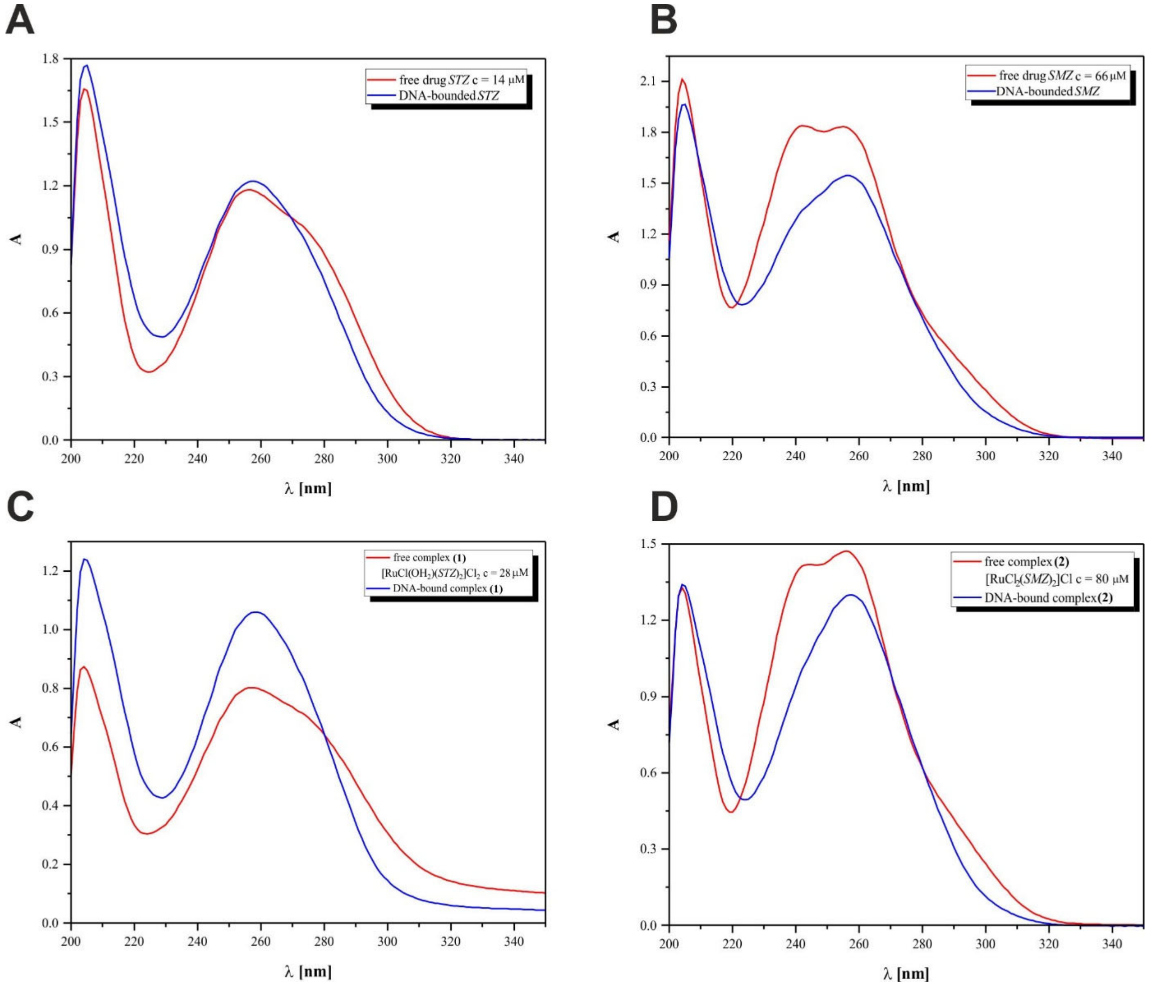

Figure S15. The spectral changes resulting from the interactions with *CT*-DNA and: (**A**) STZ, (**B**) SMZ, (**C**) Ru(III)-STZ complex **(1)**, (**D**) Ru(III)-SMZ complex **(2)**.

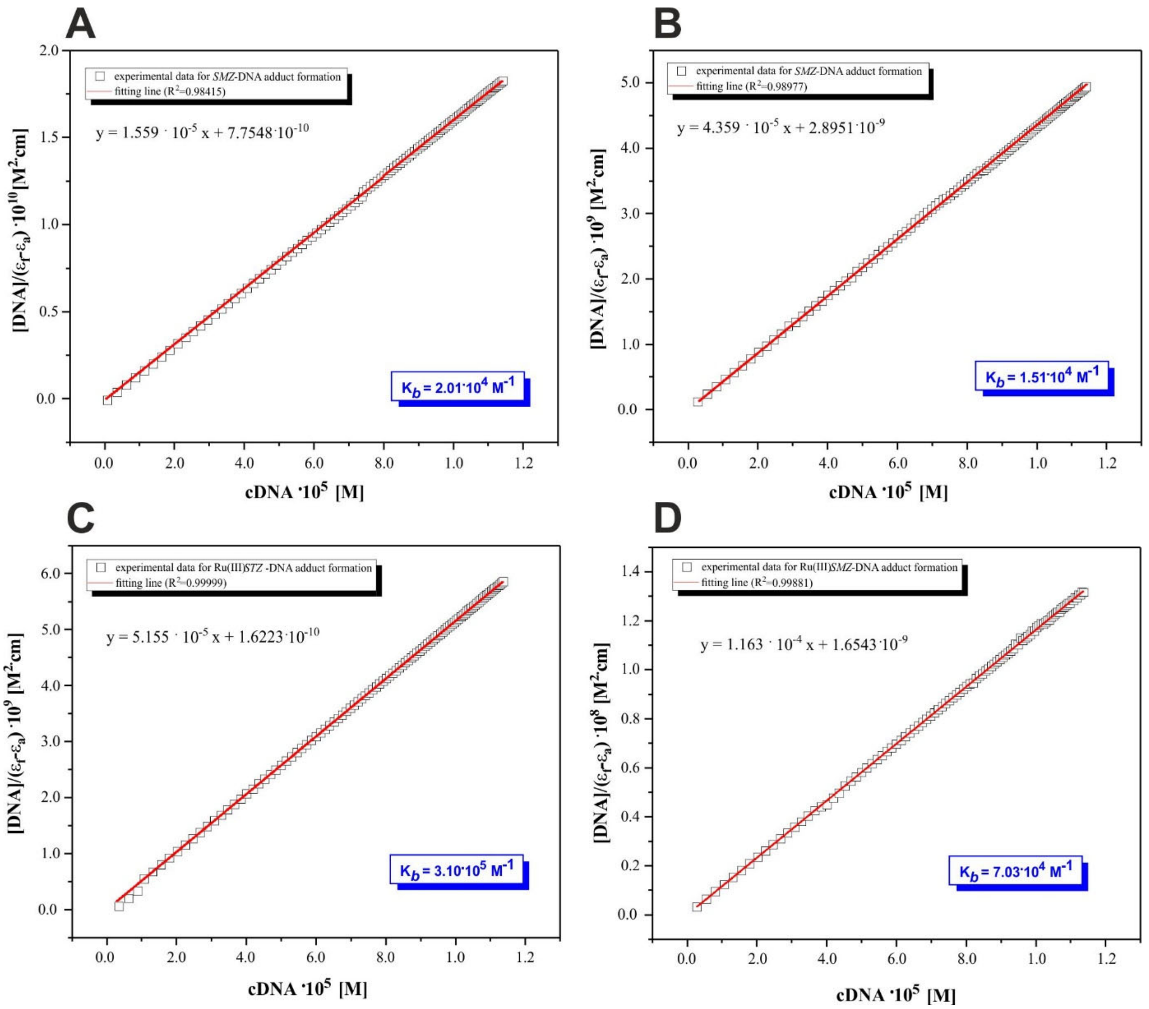

Figure S16. Plots of [DNA/(ε_f-ε_a)] *vs* [DNA] presented the affinity for DNA interactions of: (**A**) STZ, (**B**) SMZ, (**C**) Ru(III)-STZ complex **(1)**, (**D**) Ru(III)-SMZ complex **(2)**.

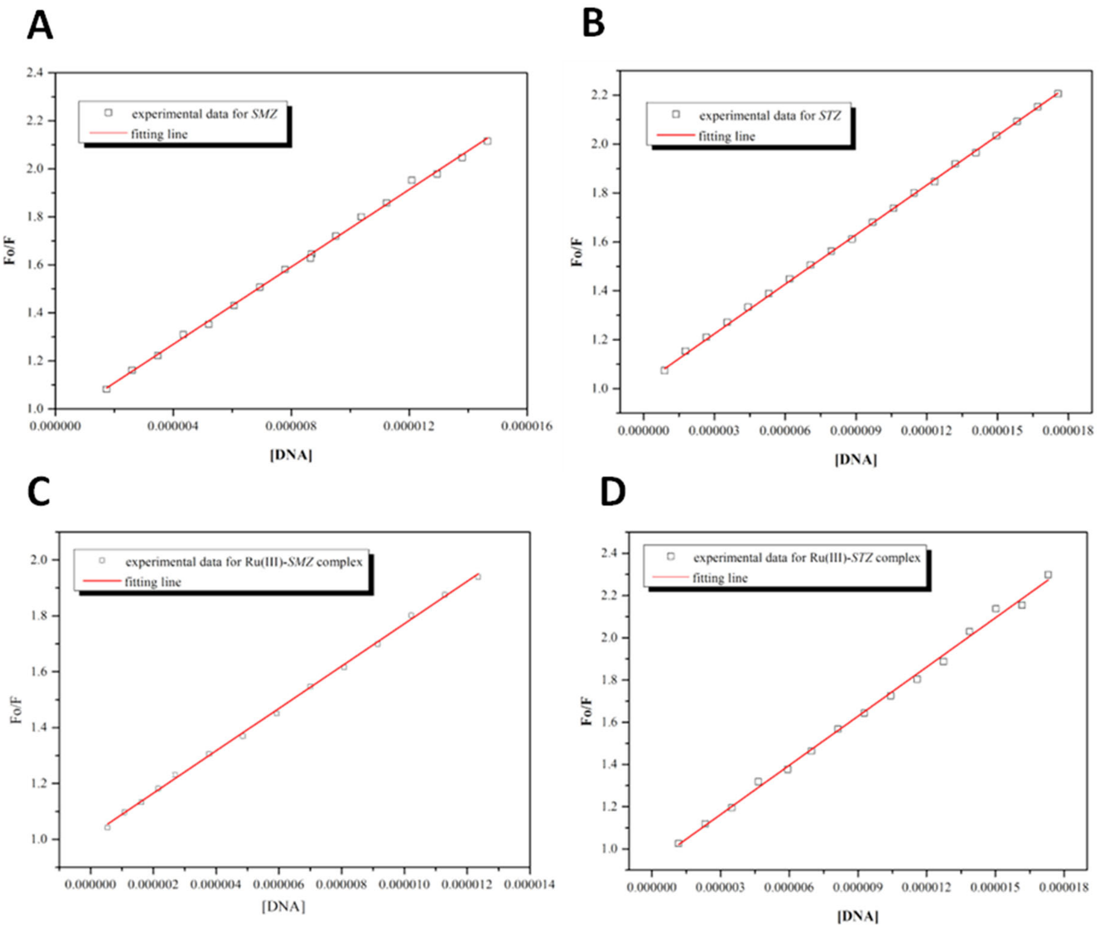

Figure S17. Stern-Volmer plots for (**A**) *SMZ*, (**B**) STZ, (**C**) Ru(III)-SMZ complex and (**D**) Ru(III)-STZ complex.

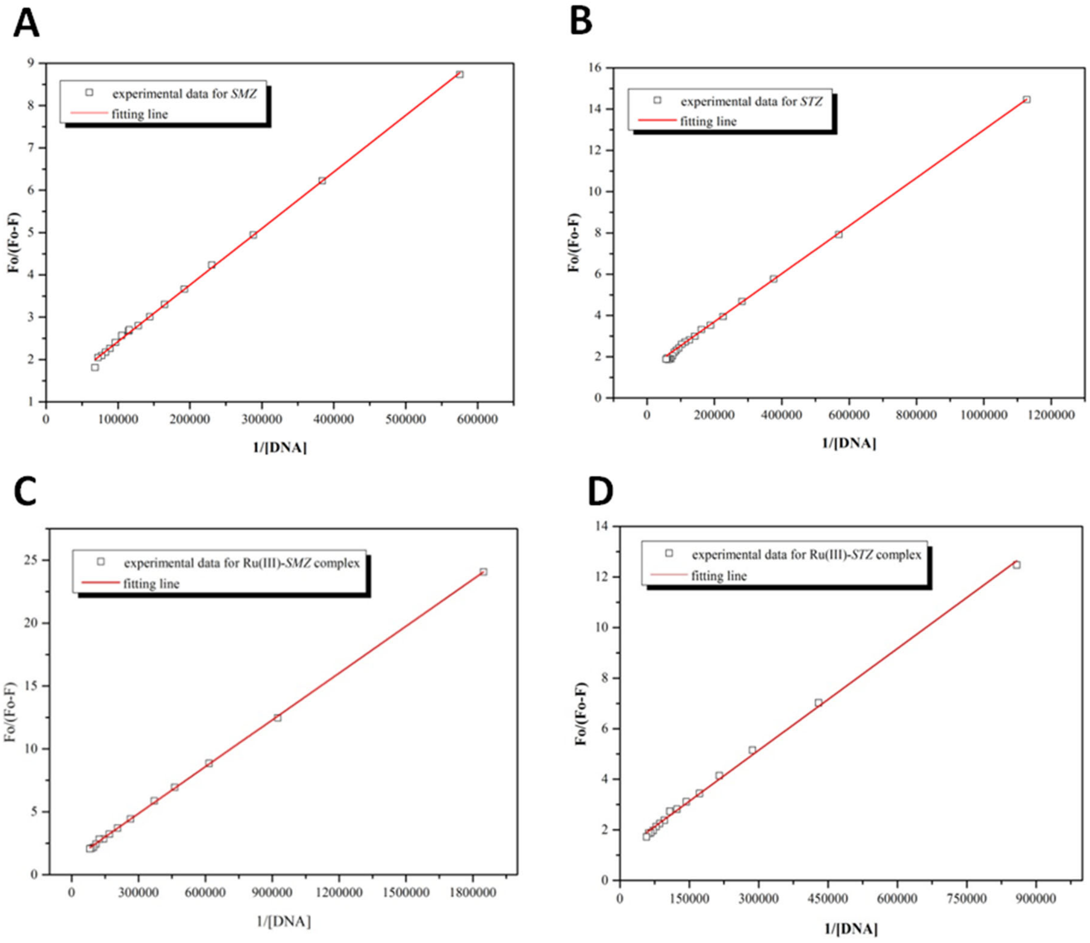

Figure S18. Lineweaver-Burk double-reciprocal curve for (**A**) SMZ, (**B**) STZ, **C.** Ru(III)-SMZ complex and **D.** Ru(III)-STZ complex.

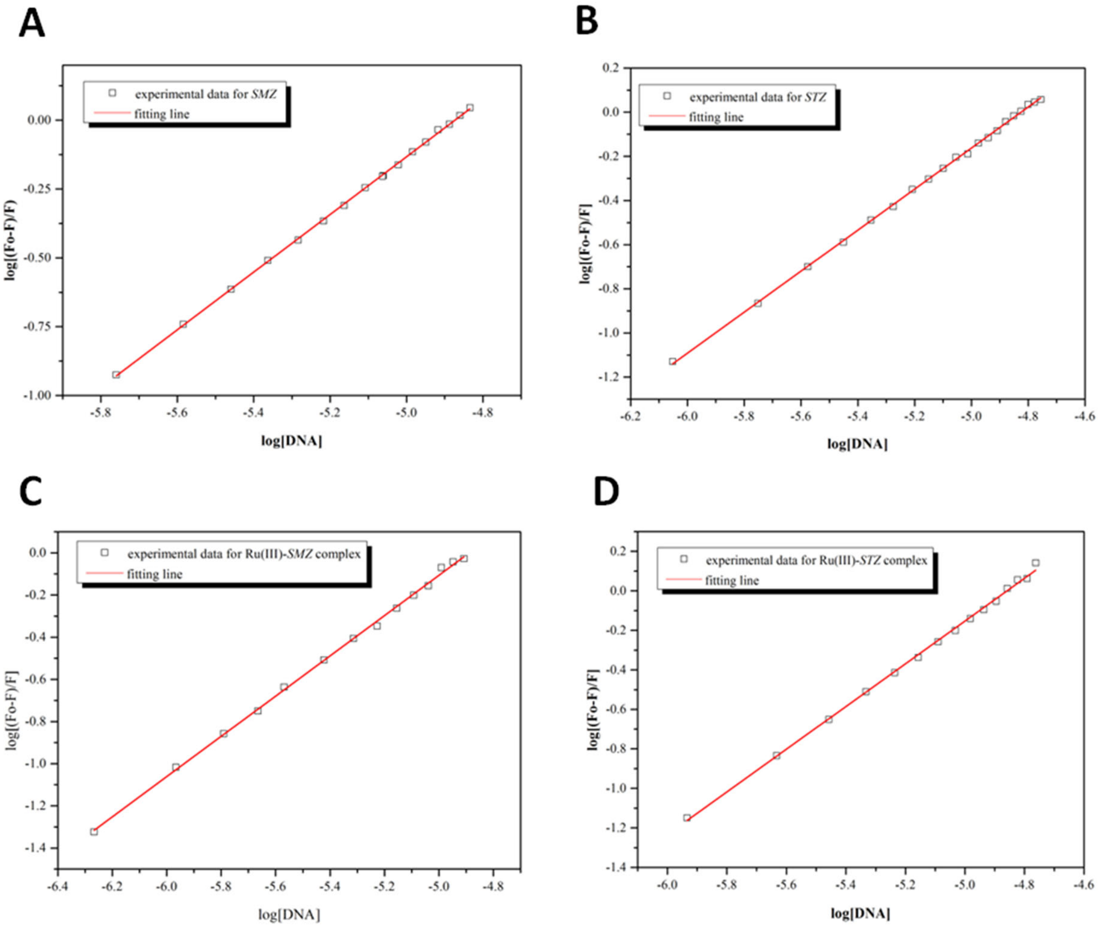

Figure S19. Scatchard's plots for (**A**) SMZ, (**B**) STZ, (**C**) Ru(III)-SMZ complex and (**D**) Ru(III)-STZ complex.

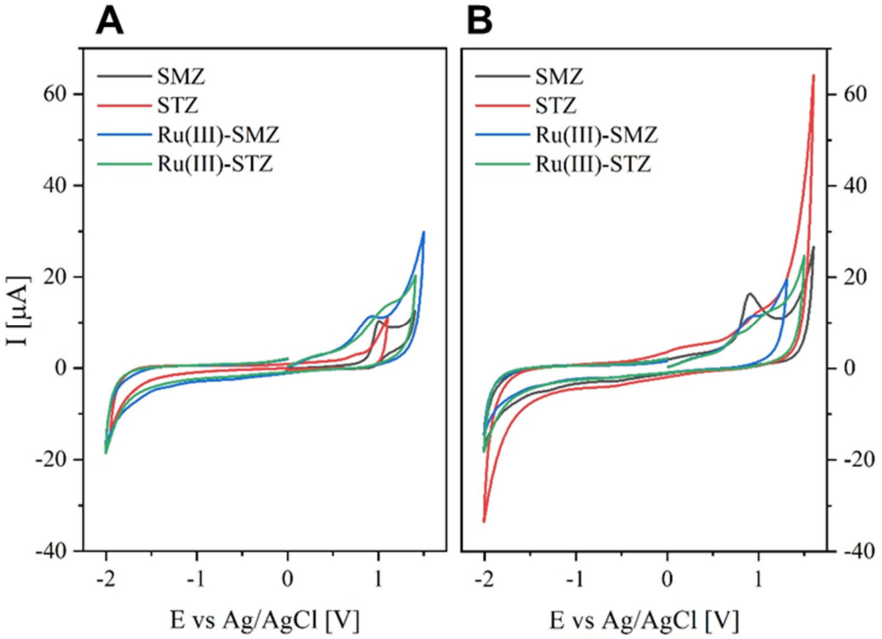

Figure S20. Voltammograms of ligands (SMZ, STZ) and their ruthenium (III) complexes (c = 10^{-1} M) registered (**A**) in water (5% DMSO) and (**B**) in Tris buffer (5% DMSO).

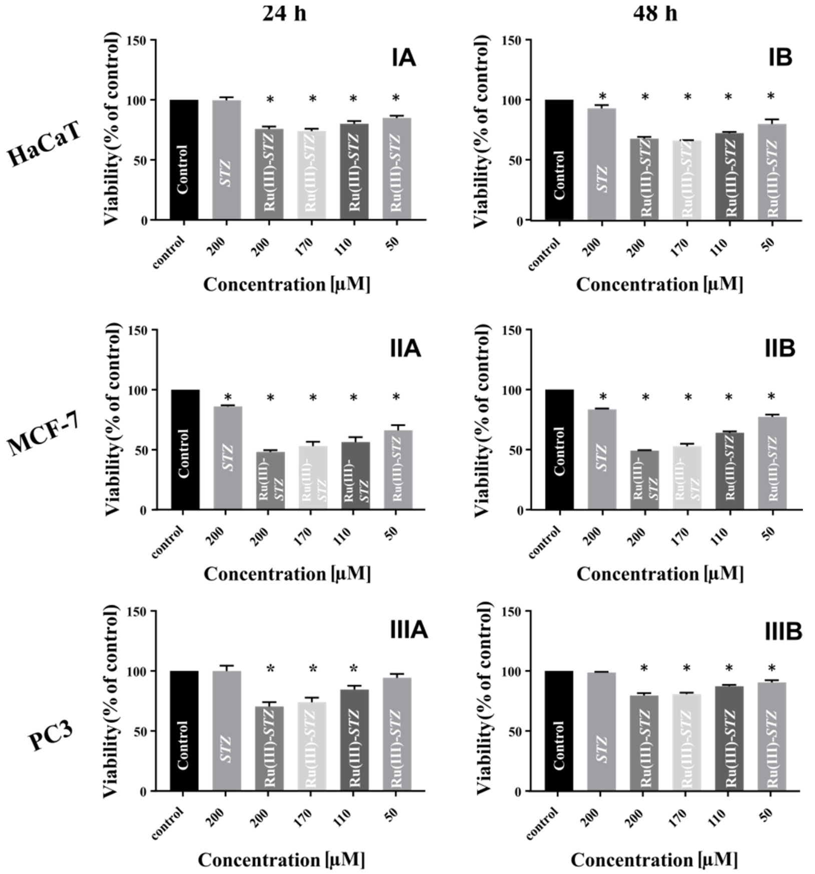

Figure S21. The viability of **I.** HaCaT, **II.** MCF-7 and **III.** PC3 cells after: (**A**) a 24 and (**B**) a 48 h treatment with STZ and Ru(III)-STZ complex in range of concentration from 0 (control) to 200 μM. Results are shown as mean ± SD of three independent experiments performed in triplicate. *statistically significant difference is present between treated cells compared with untreated cells (control).

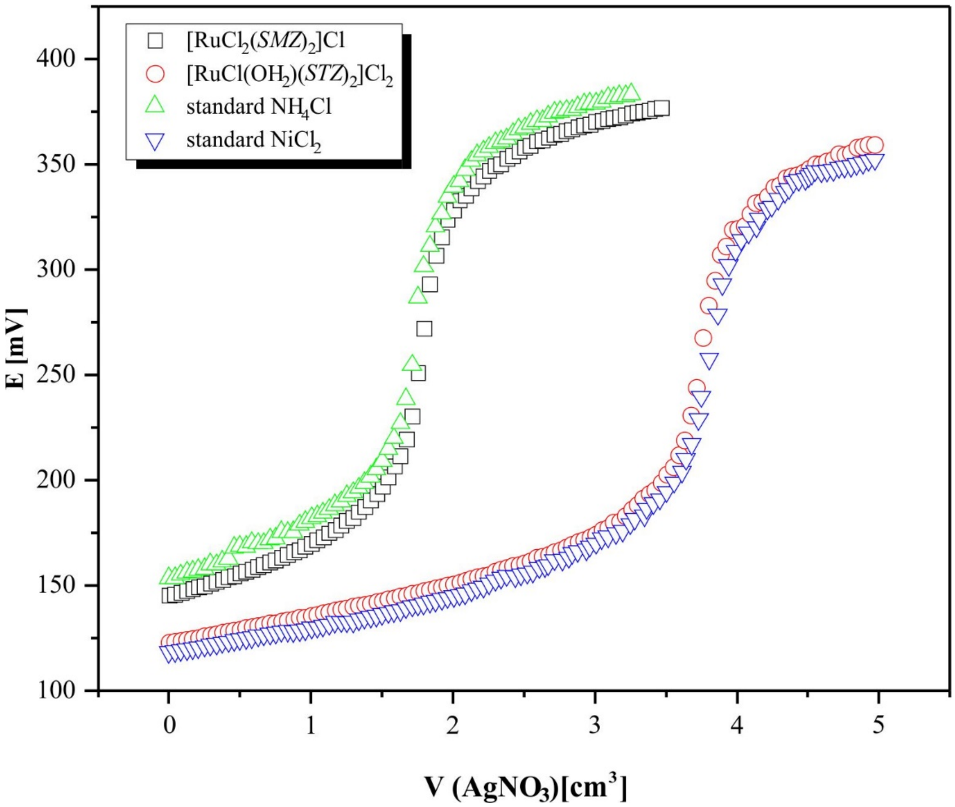

Figure S22. Precipitation titration results obtained by potentiometry with selective electrode (Cl^-). The study was performed to determine chloride ions in the formula of complexes **(1)** and **(2)** as counter ions. The standards used were NH_4Cl and $NiCl_2$. The concentration of $AgNO_3$ titrant was 5.81 mM. The titrations were made by CerkoLab microsystem with an automatic and computer controlled injector equipped with a 5 mL Hamilton syringe with a dose volume of 0.021770 mL.

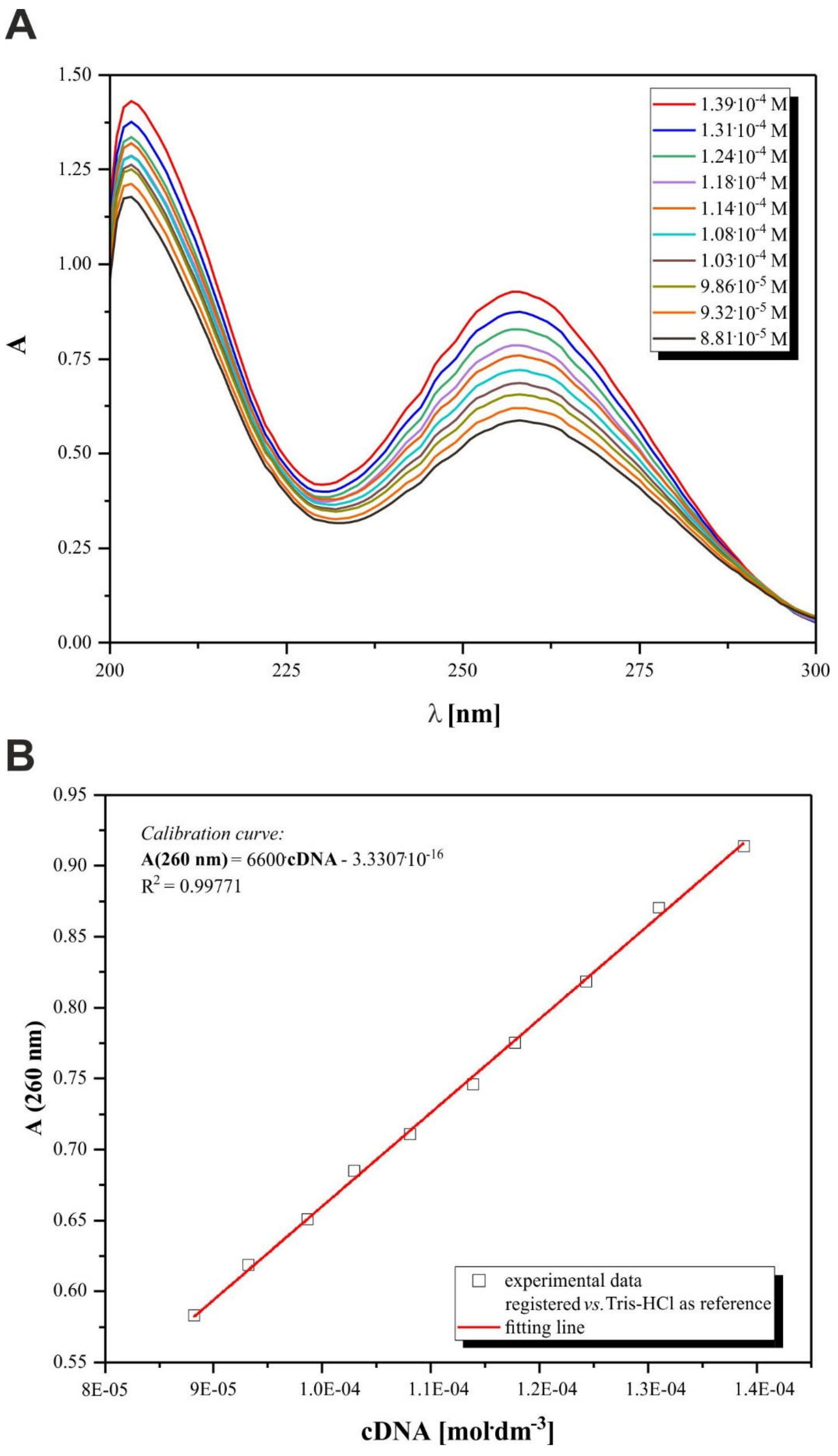

Figure S23. Electronic spectra (UV) of different DNA concentrations ($8.81 \cdot 10^{-5} \div 1.39 \cdot 10^{-4}$ M), (**B**) calibration curve for *CT*-DNA.
